# Designing Coiled Coils for Heterochiral Complexation to Enhance Binding and Enzymatic Stability

**DOI:** 10.1021/acs.biomac.4c00661

**Published:** 2024-07-09

**Authors:** Vincent P. Gray, Rachel A. Letteri

**Affiliations:** Department of Chemical Engineering, University of Virginia, Charlottesville, Virginia 22903, United States

## Abstract

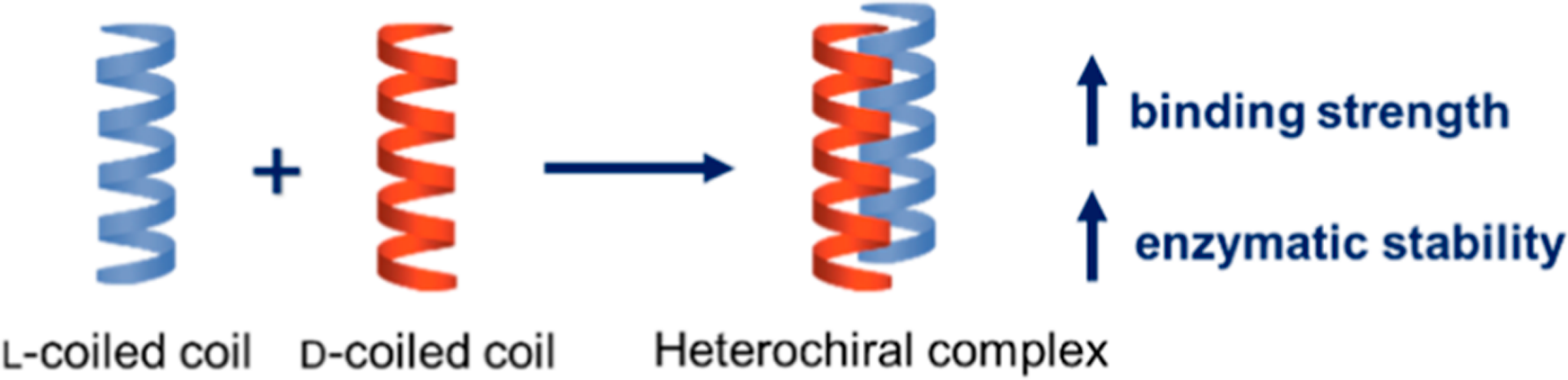

Coiled coils, commonly found in native proteins, are helical motifs important for mediating intermolecular interactions. While coiled coils are attractive for use in new therapies and biomaterials, the lack of enzymatic stability of naturally occurring l-peptides may limit their implementation in biological environments. d-peptides are of interest for biomedical applications as they are resistant to enzymatic degradation and recent reports indicate that stereochemistry–driven interactions, achieved by blending d- and l-peptides, yield access to a greater range of binding affinities and a resistance to enzymatic degradation compared to l-peptides alone. To our knowledge, this effect has not been studied in coiled coils. Here, we investigate the effects of blending heterochiral E/K coiled coils, which are a set of coiled coils widely used in biomaterials. We found that we needed to redesign the coiled coils from a repeating pattern of seven amino acids (heptad) to a repeating pattern of 11 amino acids (hendecad) to make them more amenable to heterochiral complex formation. The redesigned hendecad coiled coils form both homochiral and heterochiral complexes, where the heterochiral complexes have stronger heats of binding between the constituent peptides and are more enzymatically stable than the analogous homochiral complexes. Our results highlight the ability to design peptides to make them amenable to heterochiral complexation, so as to achieve desirable properties like increased enzymatic stability and stronger binding. Looking forward, understanding how to engineer peptides to utilize stereochemistry as a materials design tool will be important to the development of next-generation therapeutics and biomaterials.

## Introduction

**Figure 1:**
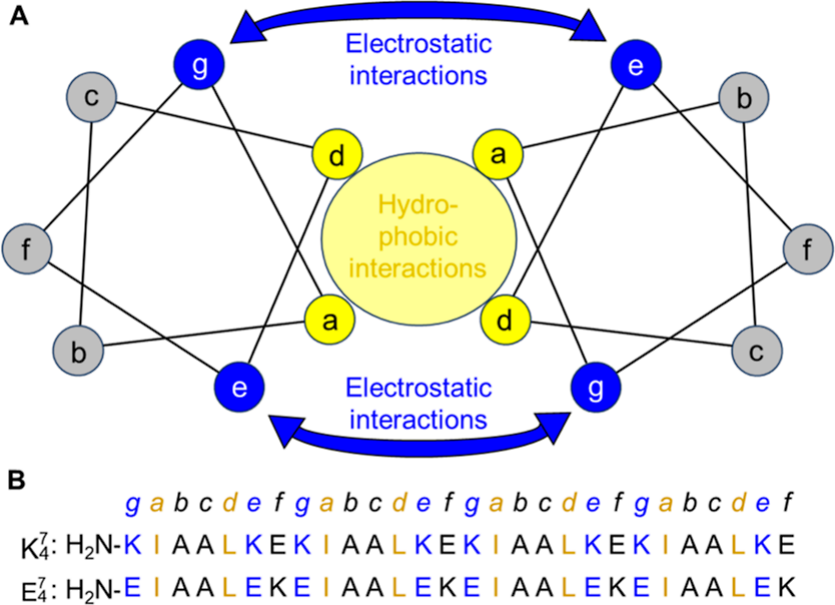
(A) Helical wheel diagram of the heptad repeat abcdefg, with hydrophobic interactions between complementary coils highlighted in yellow and electrostatic interactions between complementary coils highlighted in blue. (B) Sequences of K_4_^7^ and E_4_^7^ aligned with the heptad abcdefg registry.

Coiled coils are common helical structural motifs estimated to be found in approximately 10% of all eukaryotic proteins.^1,2^ Specifically, coiled coils mediate interactions between proteins, operating, for example, in the regulation of DNA transcription and muscle contraction.^3,4^ These functions are possible in complex biological environments as coiled coils have strong binding with a high degree of specificity.^4,5^ Coiled coil specificity and affinity are derived from a combination of hydrophobic, electrostatic, and hydrogen bonding interactions, arising from conserved regions within coiled coil sequences. Typically, coiled coils are composed of repeating patterns of seven amino acids (i.e., a heptad), labeled *abcdefg*, where amino acids in the *a* and *d* positions are hydrophobic and often those in the *e* and *g* positions are charged (Figure 1A).^3,6−9^ While the strong affinity and high specificity of coiled coils make them attractive for use in biomaterials applications,^10−16^ the lifetime of peptides in vivo is limited by poor enzymatic stability.^17−20^

One strategy to make peptide materials more stable is to alter stereochemistry, as d-amino acids resist degradation by enzymes.^21−25^ While we could certainly improve the enzymatic stability of coiled coil biomaterials by making them entirely of d-amino acids, recent reports suggest that invoking stereochemistry-directed interactions between entirely l-peptides and entirely d-peptides gives rise to properties distinct from those of naturally occurring l-peptides, including enhanced mechanics, stronger peptide–peptide interactions, and greater enzymatic stability. For example, 1:1 heterochiral blends of the d- and l-forms of the β-sheet forming peptide “MAX1” result in hydrogels with a stiffness four times greater than those formed from homochiral d- or l-MAX1.^26,27^ In another example, homochiral triple helices of the collagen-mimetic peptide (PPG)_10_ are soluble but heterochiral mixtures precipitate, a result attributed to more favorable packing interactions for the heterochiral triple helices compared to homochiral.^28^ Moreover, the enthalpy of interaction is stronger between heterochiral blends of peptides Ac-(FKFK)_2_-NH_2_ and Ac-(FEFE)_2_-NH_2_ as compared to homochiral peptide interactions.^29^ With respect to enzymatic stability, the l-form of the peptide Ac-(FKFE)_2_-NH_2_ degrades within a day upon incubation with protease, while 1:1 blends of d- and l-(FKFE)_2_ remain stable for at least 5 days, similar to the d-form of the peptide.^30^ We sought to combine the enhanced interactions and enzymatic stability of heterochiral mixtures with the specific, strong binding of coiled coils into components of next-generation biomaterials.

Interest in heterochiral assemblies of coiled coil peptides stems back to early structural considerations for proteins.^31,32^ Reports include a tetramer formed from heterochiral heptads,^33^ yet more recent work by Gellman and co-workers highlighted that heptads may not be the most ideal sequence pattern for heterochiral assembly. Crystal structures revealed that side chain interactions between hydrophobic residues on heterochiral peptides occurred in a repeating pattern of residues spaced 3, 4, and 4 residues apart, rather than with the 3,4 residue spacing typical of the heptad repeating structure. The 3,4,4 spacing is consistent with a noncanonical repeating sequence pattern of 11 amino acids (i.e., a hendecad), labeled *abcdefghijk*, where the hydrophobic amino acids are in positions *a*, *d*, and *h*.^34,35^ In this case, the hendecad structure is preferred because coiled coils of opposing stereochemistry are unable to supercoil, a correction which aligns the hydrophobic faces of the coiled coils in conventional homochiral heptads. Rather, the hydrophobic faces of hendecads align without a need for supercoiling, making them amenable to any combination of peptide stereochemistry. While these reports provide a good basis for the structural considerations of heterochiral coiled coils, the potentially unique properties of the resulting heterochiral complexes have not yet been studied.

Here, we redesign the complementary glutamic acid/lysine (E/K)-rich coiled coil sequences (Figure 1B) ubiquitously employed as components in previously reported biomaterials^10,13,14^ to promote heterochiral complexation and compare the intermolecular interactions and enzymatic stability of the resulting complexes to those of analogous homochiral coiled coils. We found that, to allow for heterochiral complexation and the possibility of accessing unique heterochiral blend properties, we needed to redesign the original heptad repeat sequences of the E/K coils as hendecad repeat sequences. The heterochiral hendecad repeat complexes exhibit higher binding strength and greater enzymatic stability than analogous all l hendecad complexes. Unlocking the benefits of stereochemistry-directed interactions in widely used biomaterial motifs such as coiled coils has the potential to greatly extend the lifetime of and tailor intermolecular interactions for next-generation materials.

## Materials and Methods

### Materials

Phosphate-buffered saline (PBS) pellets, sodium hydroxide (NaOH, 97%) pellets, acetonitrile (HPLC grade), trifluoracetic acid (TFA, 99%), dimethyl sulfoxide (99.9%), hydrochloric acid (37 wt %), dimethylformamide (DMF, ≥99.8%), diethyl ether (≥99.0%, contains butylated hydroxytoluene as inhibitor), triisopropylsilane (98%), piperidine (≥99%), 2,2′-(ethylenedioxy)diethanethiol (95%), diisopropyl carbodiimide (99%), and Proteinase K (from *Tritirachium* album) were purchased from Sigma-Aldrich. Ultrapure water (18.2 MΩ cm) was obtained from a Thermo Scientific Smart2Pure water purification system. All chemicals were used without further purification. We note that each PBS pellet, when dissolved in 200 mL of DI water yields 1× PBS (137 mM NaCl, 2.7 mM KCl, 10 mM Na_2_HPO_4_, 1.8 mM KH_2_PO_4_). To prepare 10× PBS, we dissolved each PBS pellet in 20 mL of DI water.

### Safety

No unexpected or unusually high safety hazards were encountered in this work. However, we note that TFA used in peptide cleavage/deprotection and in HPLC solvents is highly corrosive, so we neutralize any glassware or lab supplies that come into contact with TFA using a saturated solution of sodium bicarbonate.

### Coiled Coil Synthesis

Coiled coils were synthesized in-house using a CEM Corporation Liberty Blue automated, microwave-assisted solid phase peptide synthesizer via Fmoc methods on Rink amide resin SS (0.5 mmol/g substitution, 100–200 mesh, 1% divinylbenzene, Advanced ChemTech). All syntheses began with swelling the resin in dimethylformamide (DMF) then two “dummy” coupling steps, where DMF is added to the reaction vessel alone and is heated to 90 °C like a normal coupling method. These “dummy” coupling steps serve to allow the instrument to fully warm up and consistently hit the target temperature before starting the synthesis. Fmoc-protected amino acids [Fmoc-Lys(Boc)–OH, Fmoc-Ile-OH, Fmoc-Leu-OH, Fmoc-Ala-OH, and Fmoc-Glu(OBut)–OH] were used to grow the amino acid chain. Fmoc protecting group removal was performed using 20% (v/v) piperidine in DMF and coupling reactions were performed with amino acids in the presence of the coupling agents diisopropylcarbodiimide (1 M in DMF) and Oxyma Pure (1 M in DMF) at 90 °C for 2 min. The Fmoc removal and amino acid coupling steps were repeated to build the peptide from the C-terminus to the N-terminus. The peptides were cleaved from the resin and side chain protecting groups (Boc and OBut) were removed via a 3 h, room temperature reaction in a cleavage cocktail containing 92.5% (v/v) trifluoroacetic acid, 2.5% triisopropylsilane, 2.5% 2,2′-(ethylenedioxy)diethanethiol, and 2.5% water purified by reverse osmosis (RO water). The peptides were precipitated in cold ether and centrifuged (4816*g* for 5 min at 4 °C) to isolate a peptide pellet. The peptide pellets were washed once more in cold ether and centrifuged under the same conditions to reisolate peptide pellets. The pellets were dried under vacuum for 45 min before being suspended in RO water, frozen in liquid nitrogen, lyophilized, and stored as powders at −20 °C.

### Analytical-Scale High Performance Liquid Chromatography

Peptide samples were dissolved at 1–2 mg/mL in high performance liquid chromatography (HPLC) solvent (95% ultrapure water +0.1% TFA, 5% acetonitrile +0.1% TFA) and filtered through 13 mm syringe filters with 0.45 μm polytetrafluoroethylene membranes (VWR) into 2 mL vials. HPLC was performed on a Waters Alliance e2695 HPLC system with a 2998 photodiode array detector with separation achieved using an XBridge C18 reverse-phase column (4.6 × 75 mm, 3.5 μm particle size). For crude and purified peptide samples, a 1 mL/min linear gradient from 5 to 95% (v/v) acetonitrile in water +0.1% TFA over 9 min was employed to elute the peptides from the column operating at 35 °C. The only exceptions were l-E_4_^7^ and d-E_4_^7^, which were eluted on a 1 mL/min gradient from 5 to 62% (v/v) acetonitrile in water +0.1% TFA over 17 min to better separate the peaks. Elution was monitored by absorbance at 214 nm.

### Peptide Purification by Preparative-Scale HPLC

To purify peptides, 30–40 mg of peptide was dissolved in 10 mL of HPLC solvent (95% ultrapure water +0.1% TFA, 5% acetonitrile +0.1% TFA) and twice filtered through 25 mm syringe filters with 0.45 μm polytetrafluoroethylene membranes. The filtered solution was loaded into the injection loop of a Waters 2545 HPLC system with an attached 2489 photodiode array detector and Waters Fraction Collector III collection system. The sample was separated on an XBridge C18 reverse-phase column (30 × 150 mm, 5 μm particle size). The gradients used to achieve separation are listed in Table S1 below. Eluent was collected in 13 × 100 mm glass culture tubes (VWR) and the fractions of eluent that eluted from the desired product peak were combined and lyophilized. The lyophilized powders were used to obtain HPLC chromatograms and mass spectra of the purified peptides and then were stored as powders at −20 °C.

### Matrix-Assisted Laser Desorption/Ionization Time-of-Flight Mass Spectrometry

Matrix-assisted laser desorption/ionization time-of-flight mass (MALDI-TOF) mass spectrometry was used to verify the mass of the purified peptides. Samples were prepared at ∼1 mg/mL in RO water. These samples were mixed 1:1 with a solution of cyano-4-hydroxycinnamic acid (CHCA) matrix prepared at 5 mg/mL in 70% (v/v) acetonitrile in water +0.1% TFA (2 μL of sample +2 μL of CHCA solution) by pipetting up and down 6 times. Upon mixing, a 2 μL aliquot was pipetted onto a FlexiMass SR48 target plate (Shimadzu) and left at room temperature to dry. MALDI-TOF was performed on a Shimadzu 8030 MALDI-TOF using MALDI TOFMix (LaserBio Laboratories) as a calibrant.

### Isothermal Titration Calorimetry

Isothermal titration calorimetry (ITC) experiments were performed on a standard volume affinity ITC (TA Instruments). All peptide solutions were prepared at 200 μM in 1× or 10× PBS, pH corrected to 7.40 ± 0.05 by adding 1–5 μL of 1 M NaOH and degassed for 5–10 min using a TA Instruments degassing station at 400 mm Hg. The solution to be loaded into the syringe (the titrant) was used as prepared, whereas the solution to be loaded into the sample cell (the titrand) was diluted to 20 μM by adding 9 μL of 1× or 10× PBS to 1 μL of 200 μM sample. The pH of the 20 μM sample was checked and always found to be within pH 7.40 ± 0.05. The titrant and titrand pH were always checked to ensure they were with pH 7.40 ± 0.05 immediately prior to experiments to avoid substantial heat contributions from the heat of mixing of solutions with different pH. The 20 μM sample solution was also degassed before use. Titrations consisted of an initial 2 μL injection followed by 24 10 μL injections of 200 μM titrant solution injected into 1.3 mL of 20 μM titrand solution. Following an initial delay of 200 s, injections were separated by 250 s, unless otherwise noted. Experiments were performed at 25 °C with a stirring rate of 125 rpm. The reference cell was filled with 1.3 mL of degassed, deionized water that is exchanged weekly. The resulting thermograms were baseline-subtracted and heats of binding were obtained by integrating the area under each injection peak using NanoAnalyze (TA Instruments). The obtained heats of binding were then divided by moles of injectant. Heats of dilution (titrations of titrant into buffer and buffer into titrand) were analyzed similarly, and the molar heats of dilution were subtracted from the molar heats of binding to yield the final plots of kJ/mol of injectant vs mole ratio for each titration. Reported values for maximum exothermic and endothermic heats of interaction are the average of two separate experiments, with standard deviation reported.

### Coiled Coil Degradation

Degradation was monitored by HPLC. Solutions of individual coiled coils were prepared at 200 μM in 1× PBS and pH corrected to 7.40 ± 0.05 using 1–15 μL of 1 M NaOH. A stock solution of Proteinase K enzyme was prepared at 0.5 mg/mL. For each pair of peptides, 1.5 mL of each 200 μM solution was mixed, then 30 μL of 0.5 mg/mL Proteinase K was added to the 3 mL total mixed solution to obtain a final concentration of 5 μg/mL Proteinase K. Each degradation solution was stirred at 250 rpm for the duration of the experiment. For each time point, a 1 mL aliquot of degradation solution was added to an HPLC vial, a 100 μL injection was performed on HPLC, and the remaining 900 μL was returned to the bulk degradation solution. To normalize for slight shifts in elution time due to changing solvent or environmental conditions, a MATLAB script (included in SI) was used to align all K_4_^7^ (for heptad degradation) or K_3_^11^ (for hendecad degradation) peaks to the same elution time as the elution at *t* = 0 h.

### Circular Dichroism Spectroscopy

To confirm coiled coil stereochemistry and secondary structure, we measured circular dichroism (CD) spectra for individual and mixtures of coiled coils. Unless otherwise noted, samples were prepared at 100 μM in 1× PBS (pH 7.4). For measurement of coiled coil complexes, individual coiled coils were prepared at 200 μM in 1× PBS, then mixed in equal volumes. Measurements were performed on a Jasco J-1500 CD spectrophotometer at 20 °C using a 1 mm path length quartz cuvette from 250 to 200 nm at a scanning speed of 50 nm/min. In Section S4.1, we provide details on how we optimized these conditions. For temperature-dependent studies, spectra were taken at 5 °C intervals from 5 to 95 °C using a heating rate of 5 °C/min.

## Results and Discussion

Here we investigate the effects of stereochemistry–directed interactions on the affinity and stability of coiled coil complexes. Using ITC and high performance liquid chromatography-based enzymatic stability measurements, we compare the affinities and enzymatic stabilities of analogous homochiral and heterochiral coiled coils composed of either repeating heptads or hendecads. This study demonstrates the advantages of heterochiral coiled coil complexes and provides a template for modifying existing heptad coiled coils to accommodate heterochiral coiled coil formation.

### Heptad Coiled Coil Formation: Homochiral vs Heterochiral

**Figure 2:**
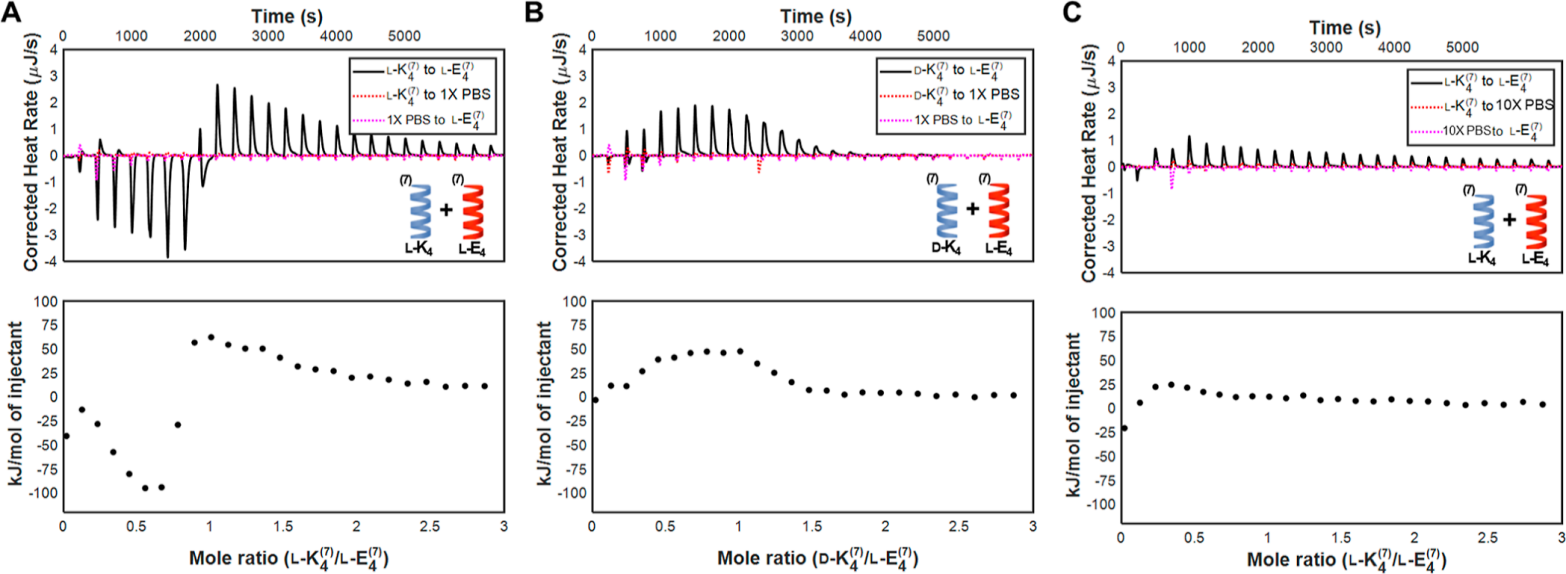
Thermograms and integrated binding heats of: (a) homochiral heptad coiled coils l-K_4_^7^ and l-E_4_^7^ in 1× PBS; (b) heterochiral heptad coiled coils d-K_4_^7^ and l-E_4_^7^ in 1× PBS; and (c) homochiral heptad coiled coils l-K_4_^7^ and l-E_4_^7^ in 10× PBS. While both exothermic and endothermic binding heats are observed for interactions between homochiral coils in 1× PBS, only endothermic binding heats are observed for interactions between heterochiral coils in 1× PBS and interactions between homochiral coils in 10× PBS.

The anion-rich coiled coil (EIAALEK)_*n*_ (E_*n*_^7^, where *n* is the number of heptad repeats and the 7 superscript indicates a heptad repeat) and cation-rich coiled coil (KIAALKE)_*n*_ (K_*n*_^7^) are known to form homochiral complexes when *n* ≥ 3.^14^ The secondary structure of these coiled coils was confirmed to be α-helical by circular dichroism (CD) spectroscopy (Figure S27). We used ITC, a label-free, solution-based technique used to study interactions between biomolecules,^36^ to assess the thermodynamics of heptad coiled coil complex formation. The homochiral titration of l-K_4_^7^/l-E_4_^7^ in 1× PBS at pH 7.4 results in heats of interaction that are initially exothermic (with a maximum binding heat of −98 ± 4 kJ/mol) until reaching a molar ratio of ∼0.8:1 l-K_4_^7^/l-E_4_^7^, after which endothermic heats of interaction (with a maximum of binding heat of 63 ± 0.6 kJ/mol) are observed (Figure 2A). This may indicate that coiled coil interactions are initially exothermic due to enthalpically favorable electrostatic interactions between complementary coils, but as binding partners are consumed, molecular rearrangements that result in the endothermic (entropically favorable) release of ordered water molecules dominate the heats of interaction.^37−39^ These thermograms are reproducible (Figure S21), yet do not fit well to single-site or multisite binding models that would allow us to obtain a binding constant to compare to other pairs. The 1:1 mixture of l-K_4_^7^ and l-E_4_^7^ yields blends that are also helical, with a stronger helical CD signal than the individual coiled coils (Figure S28A). In contrast to the homochiral titration, the heterochiral titration of d-K_4_^7^ (designed by simply switching the chirality of all amino acids in the peptide from l to d) into l-E_4_^7^ produces only endothermic binding heats smaller in magnitude (having a maximum binding heat of 48 ± 0.3 kJ/mol) than those of the corresponding homochiral pair (Figure 2B). This finding may indicate that interactions between the heterochiral heptad coils are dominated by endothermic hydrophobic interactions, with little contribution from electrostatic interactions. To test this, we repeated the homochiral titration of l-K_4_^7^ into l-E_4_^7^ in 10× PBS, where we expected the excess salt to screen electrostatic interactions. Supporting our hypothesis, we found the heats of interaction in 10× PBS to be endothermic in contrast to the exothermic and endothermic heats of interaction that we observed in 1× PBS (Figure 2C). Additionally, the CD signal of the 1:1 mixture of d-K_4_^7^ into l-E_4_^7^ is very close to zero at all wavelengths between 200 and 250 nm, indicating that the CD is simply the sum of the equimolar d- and l-coils (Figure S28C). Together, these findings indicate that simply inverting the stereochemistry of one peptide in heptad-based coiled coils disrupts complex formation to some degree, ablating any potential for stronger interactions in heterochiral complexes compared to homochiral complexes in this heptad configuration, which is consistent with previous reports of nonideality of heptad-based coiled coils for heterochiral complexation.

### Enzymatic Degradation of Heptad Coiled Coils

**Figure 3:**
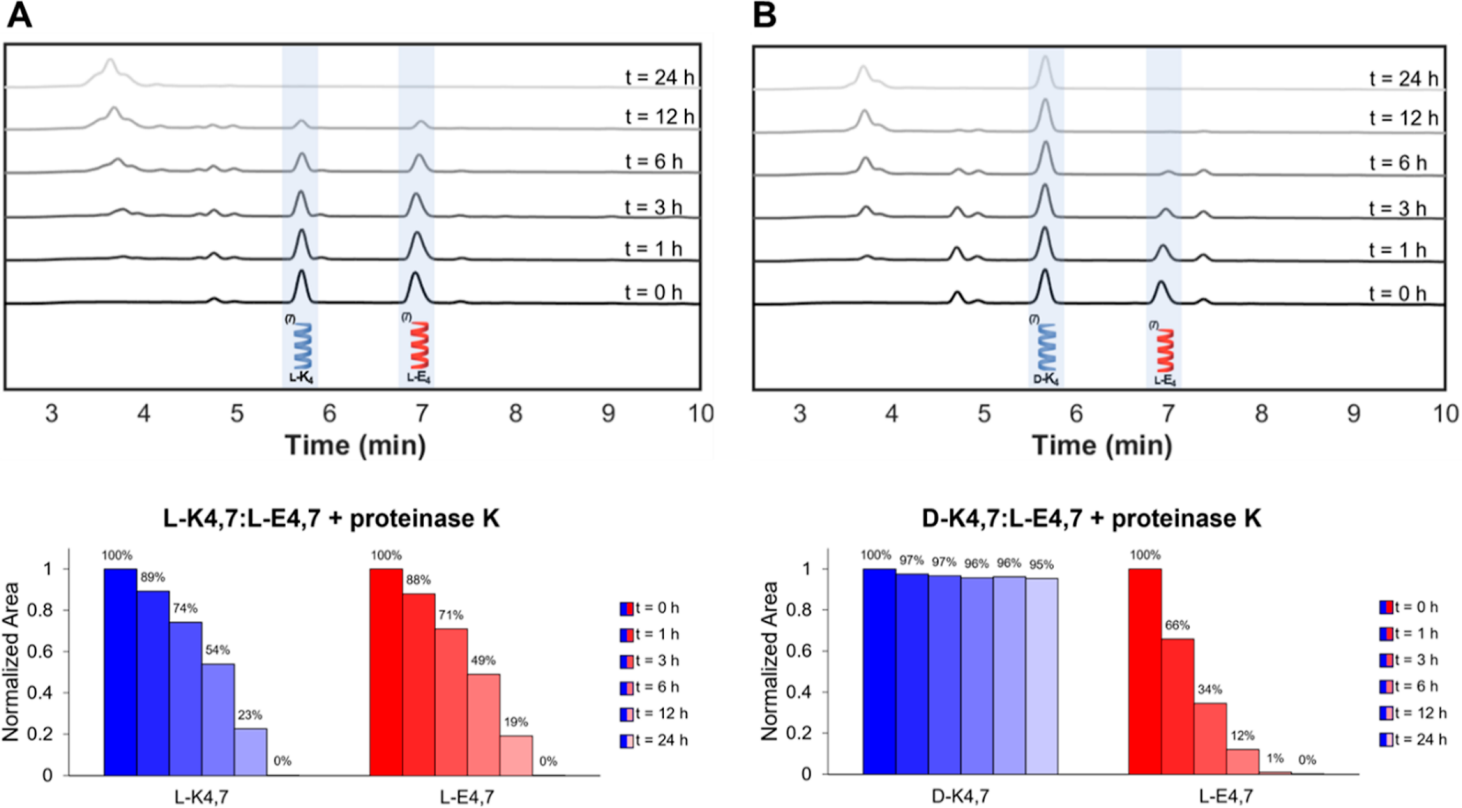
Enzymatic stability of heptad coiled coils in the presence of 5 μg/mL Proteinase K. HPLC chromatograms and percent intact K_4_^7^ and E_4_^7^ by peak area immediately after addition of and upon incubation for 1, 3, 6, 12, and 24 h with Proteinase K in (A) a homochiral blend and (B) a heterochiral blend.

While we did not observe stronger binding for heterochiral heptads, knowing that stereochemistry–directed interactions should provide both binding strength and enzymatic stability advantages, we next examined whether these heptads would exhibit more enzymatic stability in a heterochiral mixture compared to homochiral. Solutions of K_4_^7^ and E_4_^7^ (200 μM in 1× PBS at pH 7.4) were blended as homochiral (l-K_4_^7^/l-E_4_^7^) or heterochiral (d-K_4_^7^/l-E_4_^7^) mixtures. These blends were then incubated with 5 μg/mL Proteinase K, an enzyme known for its broad-spectrum activity and expected to cleave after the I, A, L, and E residues of these peptides.^40^ Immediately after adding Proteinase K, as well as after 1, 3, 6, 12, and 24 h incubations, we used high performance liquid chromatography (HPLC) to monitor the degradation of the coiled coils. In the HPLC eluent (low pH and in the presence of an organic solvent, acetonitrile), the coiled coil complex does not remain bound, resulting in two separate peaks corresponding to intact, K_4_^7^ (eluting at 5.7 min) and intact E_4_^7^ (eluting at 6.9 min). In the absence of Proteinase K, K_4_^7^, and E_4_^7^ exhibit little to no degradation over 24 h in the homochiral or heterochiral blends (Figure S31). In the presence of Proteinase K, we observe that ∼50% of the coiled coils degrade after 6 h and none of the intact coiled coils remain after just 24 h (Figure 3A). For the heterochiral blend, the d-K_4_^7^ coil experiences little to no degradation, as expected. However, the l-E_4_^7^ coil in the heterochiral complex degrades more rapidly than it did in the homochiral complex, as the peak at 6.9 min disappears completely within 12 h (Figure 3B). This is consistent with the lower heats of binding that we observe in heterochiral heptads, as the l-coil in the homochiral complex is better protected from the protease, perhaps due to shielding from the complex. Therefore, the mere presence of a d-peptide in the material is insufficient to slow enzymatic degradation. These results further motivated us to design coiled coils with a hendecad repeating pattern and repeat these experiments.

### Design of Hendecad Coiled Coils

**Figure 4:**
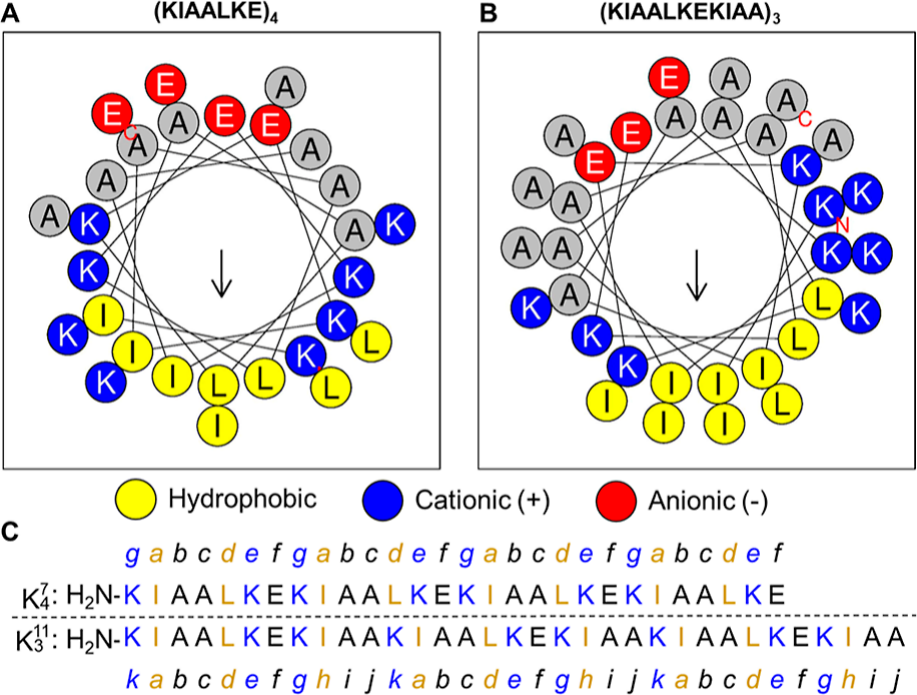
Helical wheel diagrams of; (A) K_4_^7^; and (B) K_3_^11^. Helical wheels generated using HeliQuest (https://heliquest.ipmc.cnrs.fr/). (C) Sequences of K_4_^7^ and K_3_^11^ aligned with the heptad abcdefg and hendecad abcdefghijk registers.

From Gellman and co-workers’ work, we know that a hendecad repeating structure (*abcdefghijk*), where *a*, *d*, and *h* are hydrophobic residues, is favorable for heterochiral coil formation.^34,35^ To assist with the redesign of the heptad coiled coils to a repeating hendecad structure based on E_*n*_^7^ and K_*n*_^7^, we employed helical wheel diagrams to visualize potential sequences. We observed that, as expected, the hydrophobic residues in the *a* and *d* positions of the heptad are gathered on one face of the helix, with charged residues, in the case of K_4_^7^, cationic residues specifically, flanking on either side (Figure 4A). With this knowledge, we used the same amino acids from K_*n*_^7^ to design a coiled coil with hendecad repeat structure. We first placed isoleucines and leucines in the *a*, *d*, and *h* positions, then cationic lysine residues in the *e*, *g*, and *k* positions to place them on either side of the hydrophobic face of the helix (Figure 4B), matching the placement of amino acids around the helix that we observed in the heptad repeat structure. The *b*, *c*, *i*, and *j* positions were filled with alanines, similar to the heptad structure, and the *f* position was filled with glutamic acid, serving the same role as in the heptad to provide solubility while being on the opposite side from the interacting face of the helix. For the E-rich hendecad, we used the same sequence except with all lysines exchanged for glutamic acids and vice versa. Using this newly designed hendecad-based coiled coil sequence, we again investigated complex formation and enzymatic stability of homochiral and heterochiral coils.

### Hendecad Coiled Coil Formation: Homochiral vs Heterochiral

**Figure 5:**
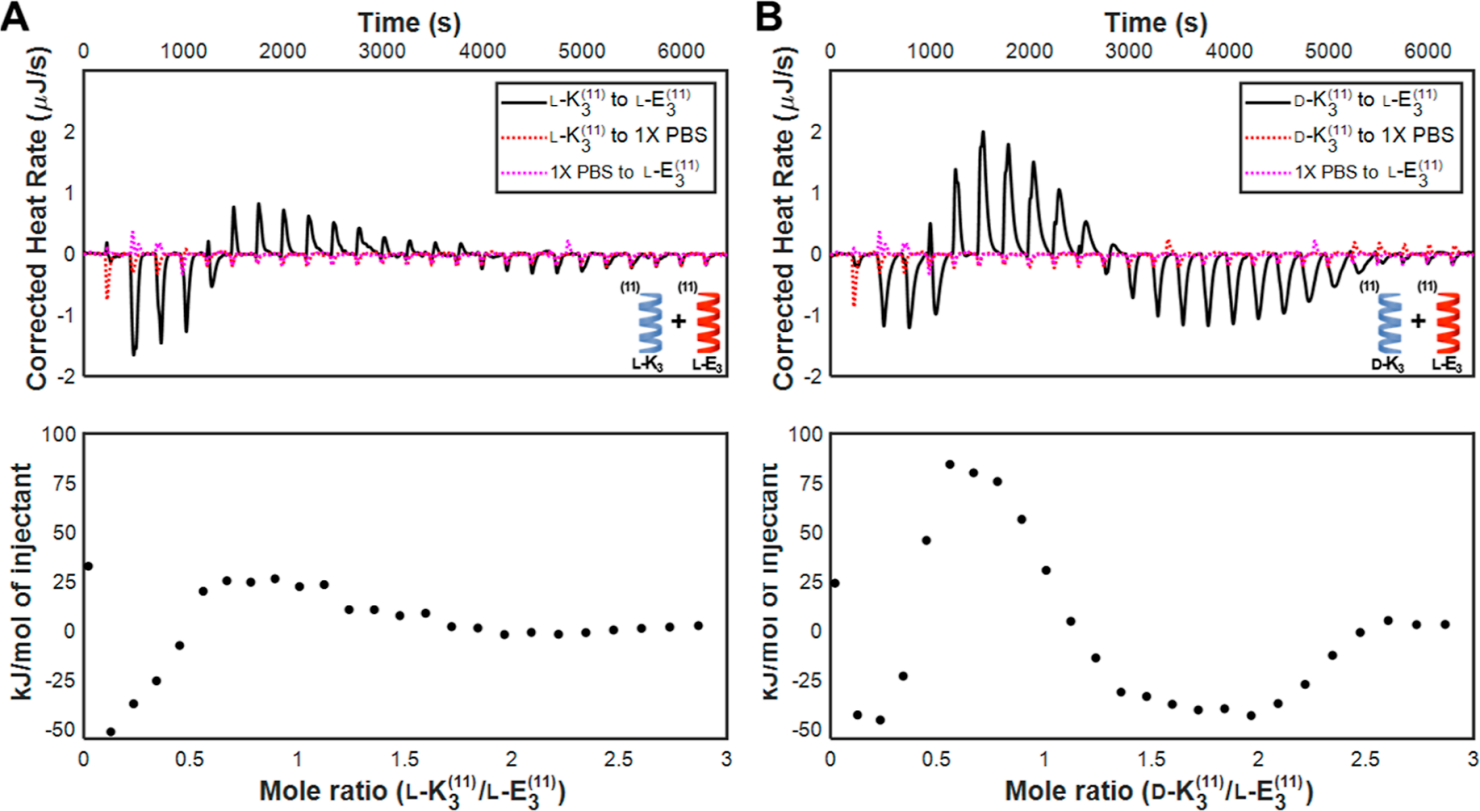
Thermograms and integrated binding heats of: (a) homochiral hendecad coiled coils l-K_3_^11^ and l-E_3_^11^ in 1× PBS; and (b) heterochiral hendecad coiled coils d-K_3_^11^ and l-E_3_^11^ in 1× PBS. Larger heats of interaction are observed for the heterochiral complex than for the homochiral complex.

To elucidate whether the change from a heptad to a hendecad repeating structure affords stronger heterochiral complex affinity compared to homochiral, we examined hendecad complex formation using ITC. We investigated hendecad coiled coils with a length of three repeats (K_3_^11^ and E_3_^11^) as they have a similar number of amino acids to the heptad coiled coils we used previously (33 amino acids for three repeats of the hendecad and 28 amino acids for four repeats of the heptad). First, we confirmed the helical secondary structure of these newly designed hendecad coiled coils using CD spectroscopy (Figure S27B). The homochiral titration of l-K_3_^11^ into l-E_3_^11^ in 1× PBS at pH 7.4 results in an interaction that is initially exothermic (with a maximum binding heat of −46 ± 8 kJ/mol) and becomes endothermic (with a maximum binding heat of 26 ± 1 kJ/mol) at a molar ratio of ∼0.5 l-K_3_^11^/l-E_3_^11^ (Figure 5A), similar to the profile observed for the homochiral interaction of the heptad coils l-K_4_^7^ and l-E_4_^7^. The binding heats then trend to zero (after subtracting the dilution heats of injectant into buffer and buffer into titrand) for all molar ratios >1.6 l-K_3_^11^/l-E_3_^11^, indicating no further interaction. Blending these coiled coils at a 1:1 ratio yields a mixture with a stronger α-helical signal by CD spectroscopy than either individual coiled coil (Figure S28B). The heterochiral titration also begins with exothermic binding heats that become endothermic, but this titration has a second exothermically dominated domain at molar ratios >1.2 d-K_3_^11^/l-E_3_^11^ and the binding heats do not trend to zero until molar ratios >2.5 d-K_3_^11^/l-E_3_^11^ (Figure 5B). While the maximum exothermic binding heats for the homochiral titration and the two exothermic domains of the heterochiral interaction are similar in magnitude (−46 ± 8 kJ/mol for the homochiral interaction and −46 ± 1 kJ/mol and −44 ± 0.7 kJ/mol for the first and second exothermic domains of the heterochiral interaction), the maximum endothermic binding heats for the heterochiral interaction are much greater than the homochiral interaction (26 ± 0.7 kJ/mol for the homochiral titration and 88 ± 5 kJ/mol for the heterochiral titration). This indicates a stronger binding affinity for the heterochiral interaction of these hendecad coiled coils consistent with what has been previously reported for Ac-(FKFE)_2_.^29^ Similarly to heterochiral heptad coiled coils, the CD signal for 1:1 d-K_3_^11^/l-E_3_^11^ (Figure S28D) is close to zero for the wavelength range from 200 to 250 nm, due to opposing stereochemistries of the equimolar blend of peptides.

### Enzymatic Degradation of Hendecad Coiled Coils

**Figure 6:**
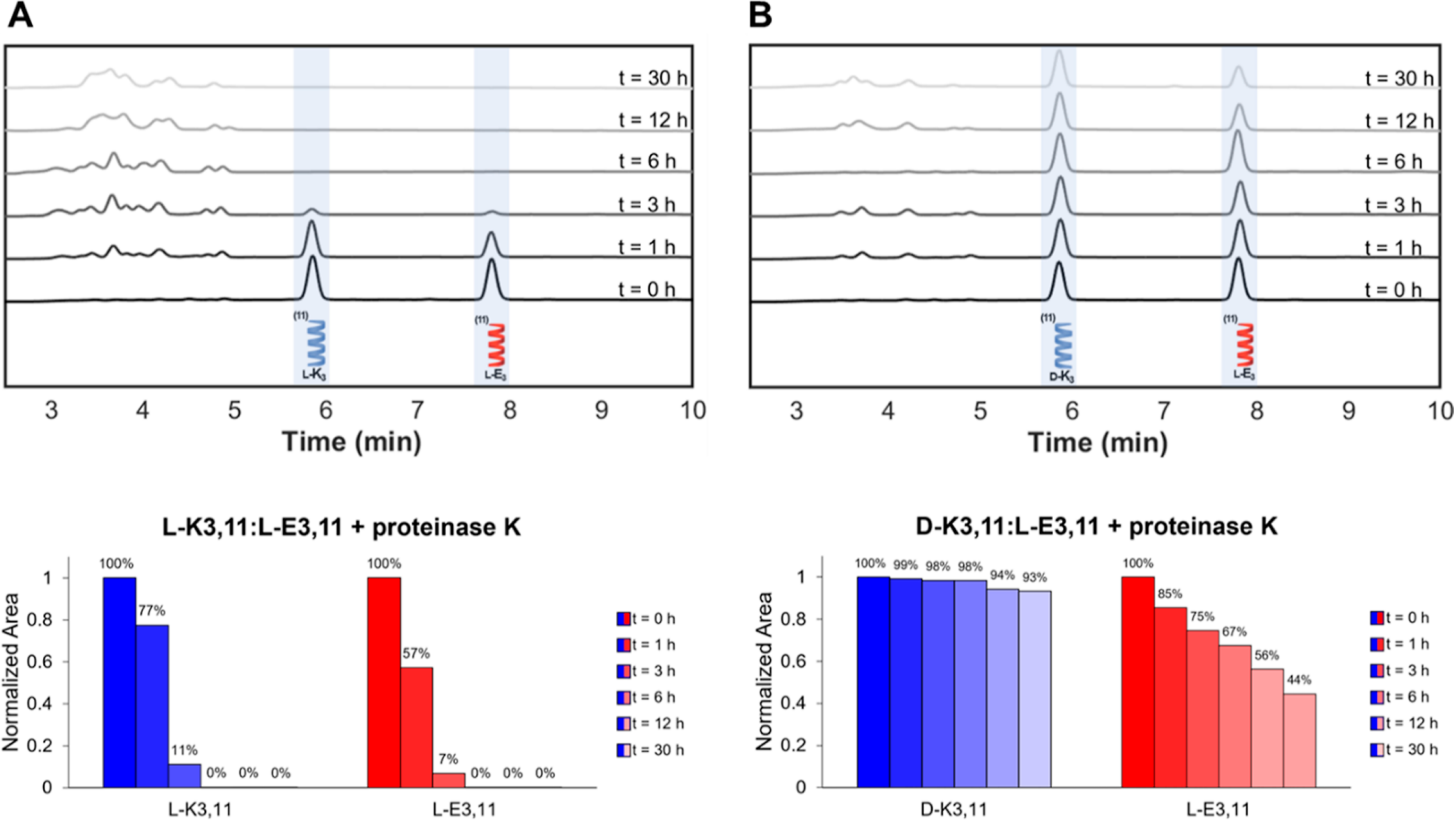
Enzymatic stability of hendecad coiled coils in the presence of 5 μg/mL Proteinase K. HPLC chromatograms and percent intact K_3_^11^ and E_3_^11^ by peak area immediately after addition of and upon incubation for 1, 3, 6, 12, and 30 h with Proteinase K in (A) a homochiral blend and (B) a heterochiral blend.

Encouraged by the stronger binding affinity we observed for heterochiral hendecad coiled coils compared to homochiral, we next investigated the enzymatic stability of our designed hendecad coiled coils. We incubated solutions of homochiral (l-K_3_^11^/l-E_3_^11^) or heterochiral (d-K_3_^11^/l-E_3_^11^) hendecad coiled coils with 5 μg/mL Proteinase K as we did for the heptad coiled coils (200 μM each in 1× PBS at pH 7.4). Similar to the heptad coiled coils, the hendecad coiled coils elute as two separate peaks, one for K_3_^11^ (eluting at 5.9 min) and one for E_3_^11^ (eluting at 7.9 min). When incubated in buffer alone without Proteinase K, both the homochiral and heterochiral complexes remain stable over 30 h (Figure S32). In the presence of Proteinase K, both l-K_3_^11^ and l-E_3_^11^ in the homochiral complex degrade completely in under 6 h, with only 11 and 7% of the peak area remaining for each, respectively, after 3 h (Figure 6A). On the other hand, 44% of the l-E_3_^11^ in the heterochiral complex remains after 30 h of incubation (Figure 6B), and we found that even after 7 days of incubation, l-E_3_^11^ did not completely degrade as 23% of the peptide still remains (Figure S33). As expected, the d-K_3_^11^ in the heterochiral complex remains largely stable over the 30 h incubation with Proteinase K. These results demonstrate that forming a heterochiral complex using our designed hendecad coiled coils is an effective strategy to protect a coiled coil in the natural l stereochemistry from degradation.

## Conclusions

This work highlights that peptides can be rationally designed to undergo heterochiral interactions and thereby unlock a larger range of binding affinities and better control over enzymatic stability. Experiments with coiled coils featuring the canonical heptad repeat pattern reveal that they bind stronger as homochiral compared to heterochiral mixtures, but with limited enzymatic stability. Redesigning these heptad coiled coils into noncanonical hendecad repeat patterns enables complexation from both homochiral and heterochiral mixtures, with greater binding strength and enzymatic stability observed for the latter. The consistency between the binding heats observed in ITC and the enzymatic degradation profiles from HPLC throughout the manuscript corroborated the ITC results, despite the ITC data not fitting to binding models. We observed that, in cases where the binding heats for one complex were smaller than another, the l-peptides in that complex degraded more quickly in the presence of enzyme, suggesting that such enzymatic stability measurements are a useful tool to assess intermolecular interactions. Going forward, while the design rules for homochiral coiled coils with heptad repeating patterns are relatively well-known, continuing to correlate peptide sequence design in both homochiral and heterochiral mixtures of hendecad coiled coils to properties of the resulting complexes will provide important insights that enrich our molecular toolkit for engineering tunable materials.

## Supporting Information Available

Materials, experimental details, peptide characterization, additional ITC thermograms, circular dichroism spectroscopy, and additional HPLC degradation experiments (PDF)

## Abbreviations

ITC: isothermal titration calorimetry
HPLC: high performance liquid chromatography
PBS: phosphate buffered saline
