# Supplementary material for: Designing Coiled Coils for Heterochiral Complexation to Enhance Binding and Enzymatic Stability
Source: Biomacromolecules. 2024 Jul 9;25(8):5273–80. doi: 10.1021/acs.biomac.4c00661 (PMC11323006; doi:10.1021/acs.biomac.4c00661)
Supplement: Supplementary file 1 — bm4c00661_si_001.pdf [file bm4c00661_si_001.pdf]

Supporting Information for

Designing Coiled Coils for Heterochiral Complexation to Enhance Binding and Enzymatic Stability

Vincent P. Gray[a] and Rachel A. Letteri[a*]

[a]Department of Chemical Engineering, University of Virginia, Charlottesville, Virginia 22903, United States
[*]Email: rl2qm@virginia.edu

Table of Contents

1. **Peptide Characterization** 2
1.1 L-K_4^7 4
1.2 D-K_4^7 6
1.3 L-E_4^7 9
1.4 L-K_3^{11} 11
1.5 D-K_3^{11} 13
1.6 L-E_3^{11} 15
1.7 MALDI of CHCA matrix 17
2. **Additional ITC thermograms** 17
2.1 Replicate of L-K_4^7 titrated into L-E_4^7 17
2.2 Replicate of D-K_4^7 titrated into L-E_4^7 18
2.3 Replicate of L-K_3^{11} titrated into L-E_3^{11} 19
3. **CD spectra of coiled coils** 20
3.1 Determination of CD spectroscopy conditions 20
3.2 CD spectroscopy of L-K_4^7, L-E_4^7, D-K_4^7, L-K_3^{11}, L-E_3^{11}, and D-K_3^{11} 22
3.3 CD spectroscopy of blended coiled coils 22
3.4 Thermal stability of L-K_4^7: L-E_4^7, D-K_4^7: L-E_4^7, L-K_3^{11} : L-E_3^{11}, and D-K_3^{11} : L-E_3^{11} as measured by CD spectroscopy as a function of temperature 23
4. **Additional degradation data** 25
4.1 Degradation of coiled coils in the absence of Proteinase K 25
4.2 Extended degradation of D-K_3^{11} : L-E_3^{11} upon incubation with Proteinase K 26
5. **MATLAB script to normalize elution time for degradation data** 27

1. Peptide Characterization

The coiled coil peptides used in this study were purified by preparative-scale HPLC using the binary gradients listed in Table S1 below. The following figures show chromatograms of crude and purified peptides, as well as MALDI-TOF mass spectra of each purified peptide.

Table S1. List of elution conditions for preparative-scale HPLC purification of coiled coils.

**Peptide**	**Binary gradient (Solvent A = water + 0.1% TFA, Solvent B = acetonitrile + 0.1% TFA)**

L-K_4^7	5 to 30% B from 2.22 to 4 min, 30 to 45% B from 4 to 32 min
D-K_4^7	5 to 30% B from 2.22 to 4 min, 30 to 45% B from 4 to 32 min
L-E_4^7	5 to 95% B from 4 to 32 min
L-K_3^{11}	5 to 30% B from 2.22 to 4 min, 30 to 60% B from 4 to 25 min
D-K_3^{11}	5 to 30% B from 2.22 to 4 min, 30 to 60% B from 4 to 25 min
L-E_3^{11}	5 to 30% B by 2.22 min, 30 to 95% B from 2.22 to 22 min
D-E_4^7	5 to 95% B from 4 to 32 min

1.1 L-K_4^7

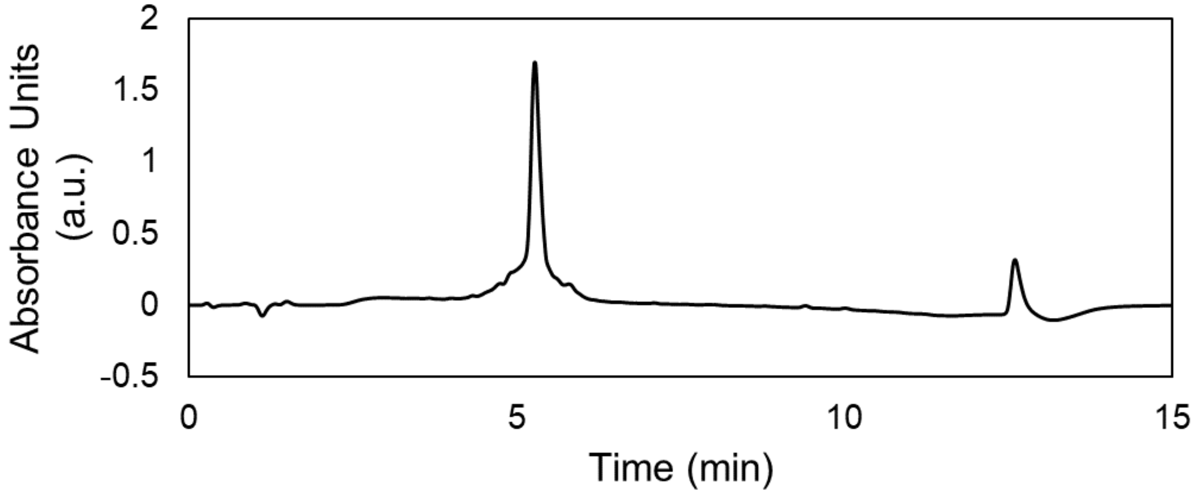

Figure S1. Analytical HPLC chromatogram of crude L-K_4^7. The peptide was eluted on a linear AB gradient from 5% to 95% B (v/v) over 9 minutes, where A is ultrapure water + 0.1% TFA and B is acetonitrile + 0.1% TFA.

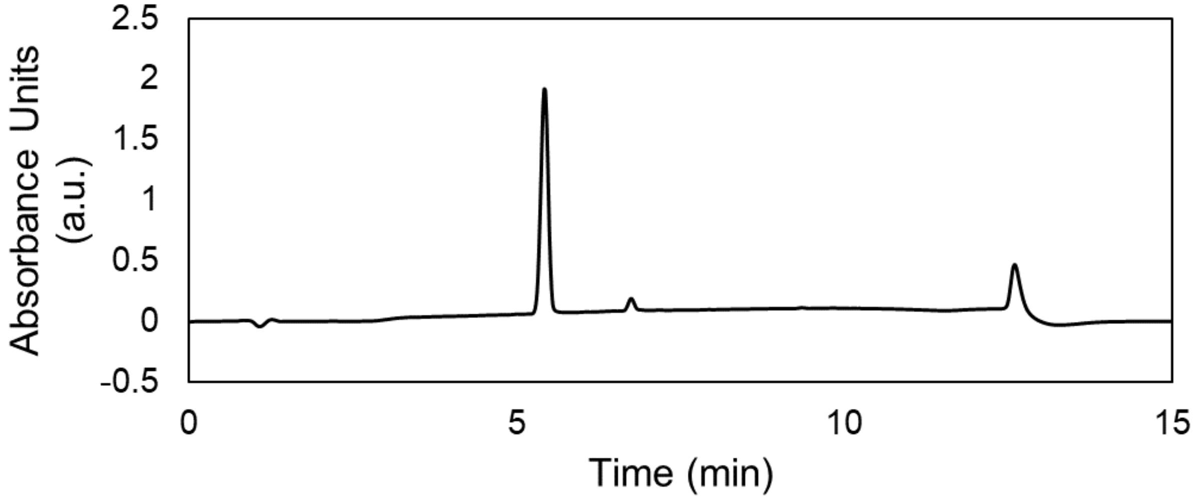

Figure S2. Analytical HPLC chromatogram of purified L-K_4^7. The primary peak corresponding to the purified peptide accounts for >96% of the total peak area. The peptide was eluted on a linear AB gradient from 5% to 95% B (v/v) over 9 minutes, where A is ultrapure water + 0.1% TFA and B is acetonitrile + 0.1% TFA.

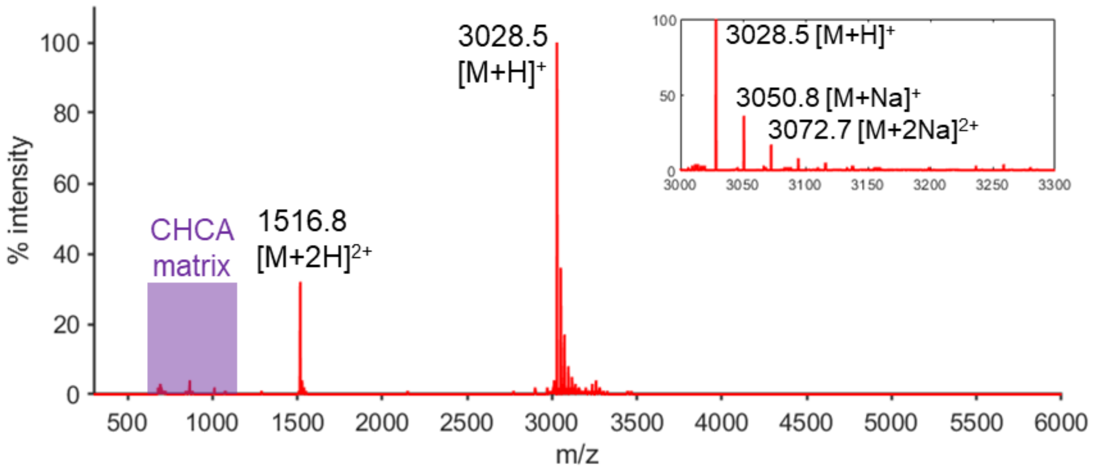

Figure S3. MALDI-TOF mass spectrum of purified L-K_4^7.

1.2 D-K_4^7

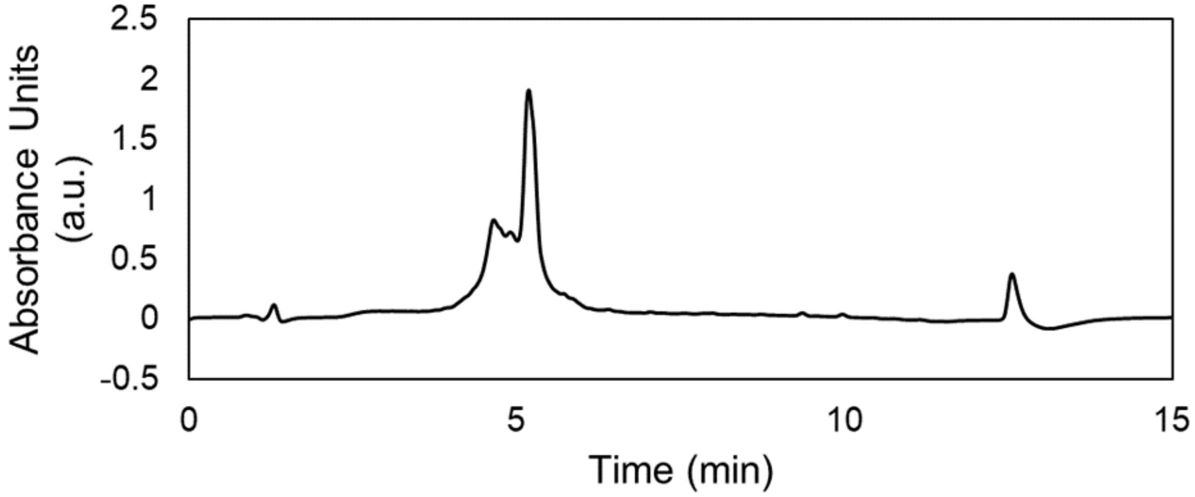

Figure S4. Analytical HPLC chromatogram of crude D-K_4^7. The peptide was eluted on a linear AB gradient from 5% to 95% B (v/v) over 9 minutes, where A is ultrapure water + 0.1% TFA and B is acetonitrile + 0.1% TFA.

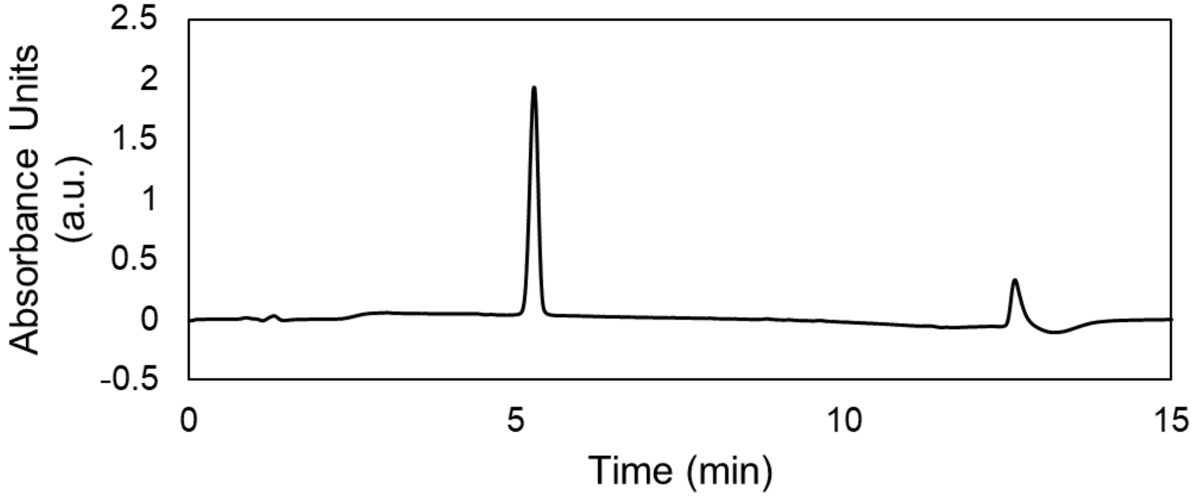

Figure S5. Analytical HPLC chromatogram of purified D-K_4^7. The primary peak corresponding to the purified peptide accounts for >99% of the total peak area. The peptide was eluted on a linear AB gradient from 5% to 95% B (v/v) over 9 minutes, where A is ultrapure water + 0.1% TFA and B is acetonitrile + 0.1% TFA.

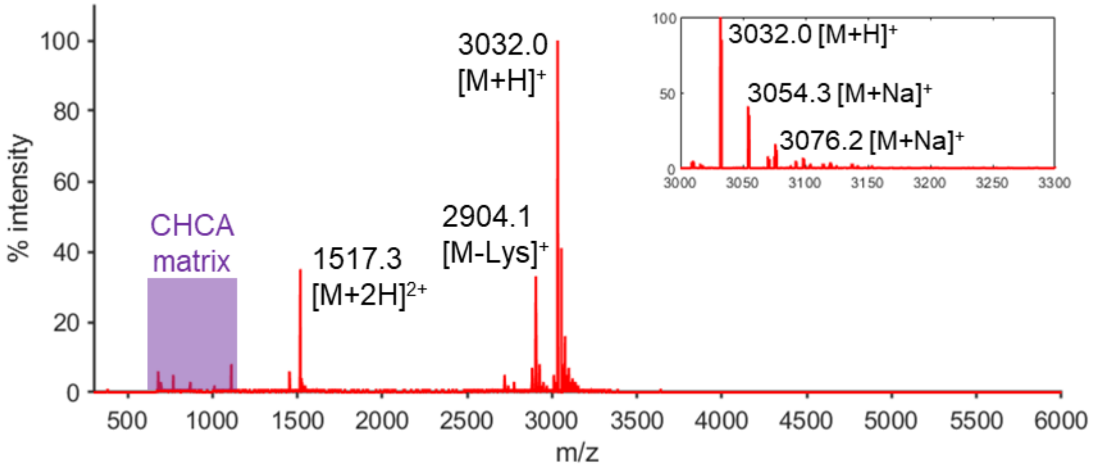

Figure S6. MALDI-TOF mass spectrum of purified D-K_4^7.

We note that the MALDI mass spectrum shows a Lys deletion. However, we see only one peak for D-K_4^7 in the analytical HPLC chromatogram for the purified peptide. Either the deletion is such a minor product that it is not detected on HPLC (which, despite the size of the peak in the mass spectrum, is possible because MALDI-TOF does not always convey relative abundance), or the deletion is not resolved from the main peptide peak under these HPLC conditions. Regardless, we used unpurified peptides to perform a titration of D-K_4^7 into L-E_4^7 (**Figure S7**), which yielded a thermogram and integrated heats of interaction that are very similar to the same titration performed with purified peptides (**Figure 2** and **Figure S21**). Therefore, even if the deletion is present in the purified D-K_4^7, it is unlikely to have affected the conclusions drawn from titrations using this peptide.

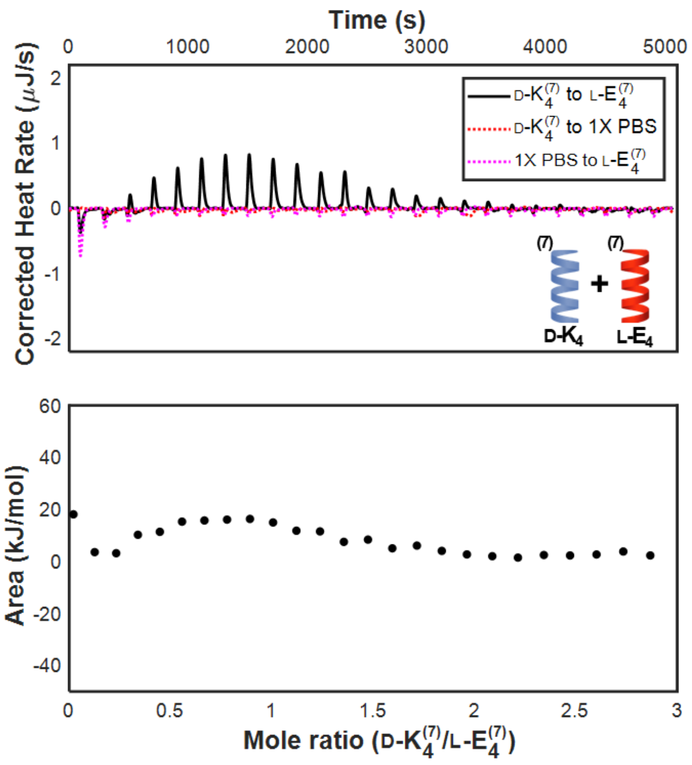

Figure S7. Thermogram and integrated binding heat from the titration of unpurified D-K_4^7 into L-E_4^7 at pH 7.4 in 1X PBS.

1.3 L-E_4^7

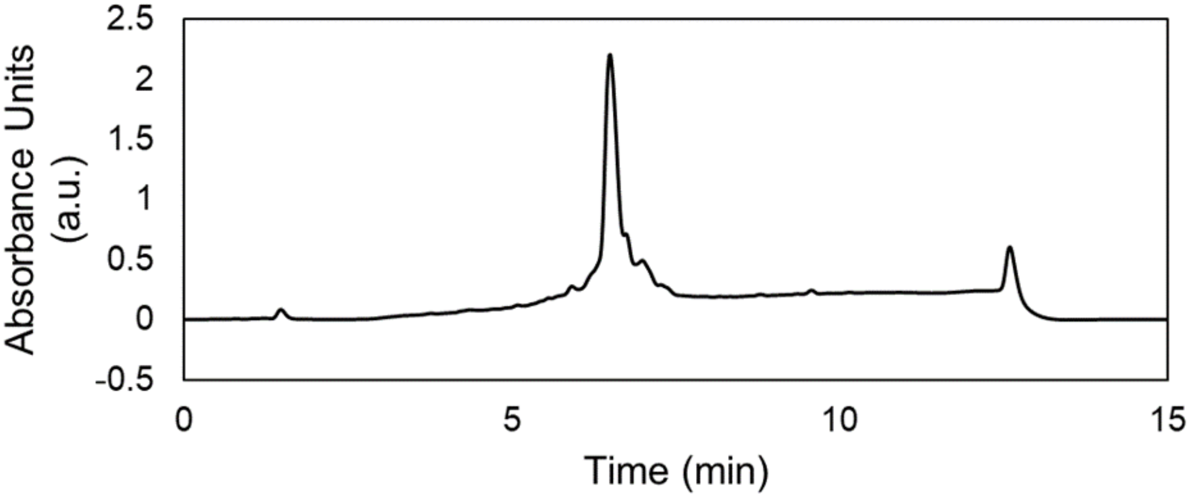

Figure S8. Analytical HPLC chromatogram of crude L-E_4^7. The peptide was eluted on a linear AB gradient from 5% to 95% B (v/v) over 9 minutes, where A is ultrapure water + 0.1% TFA and B is acetonitrile + 0.1% TFA.

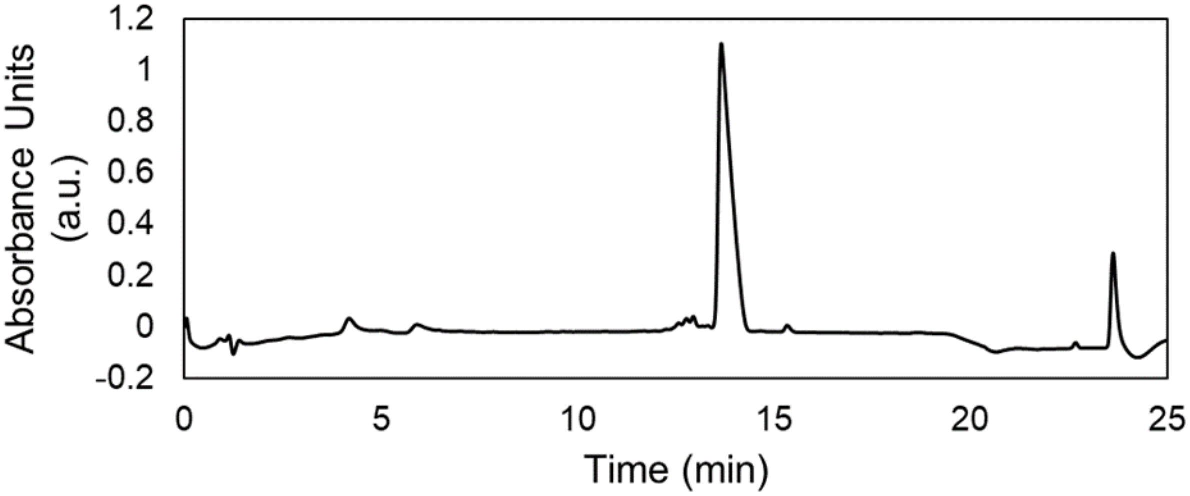

Figure S9. Analytical HPLC chromatogram of purified L-E_4^7. The primary peak corresponding to the purified peptide accounts for >93% of the total peak area. The peptide was eluted on a linear AB gradient from 5% to 62% B (v/v) over 17 minutes, where A is ultrapure water + 0.1% TFA and B is acetonitrile + 0.1% TFA.

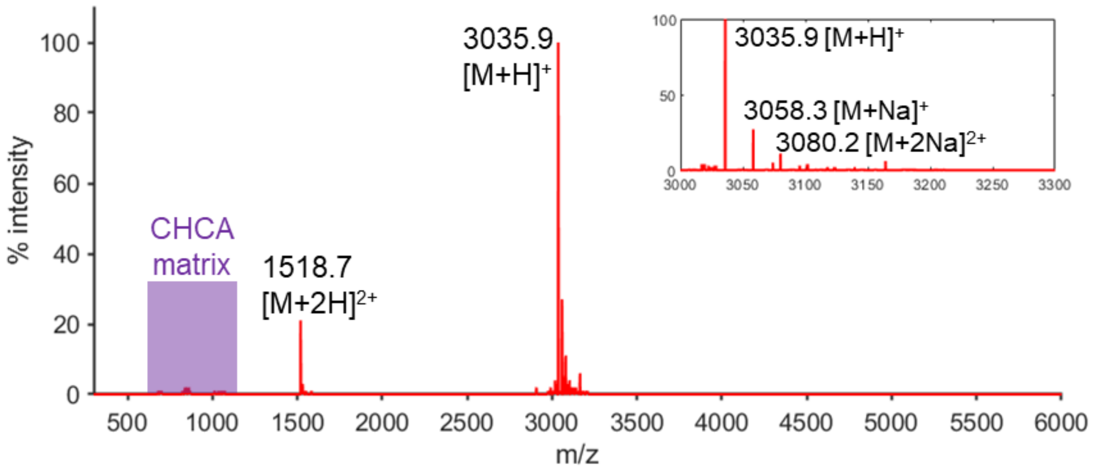

Figure S10. MALDI-TOF mass spectrum of purified L-E_4^7.

1.4 L-K_3^{11}

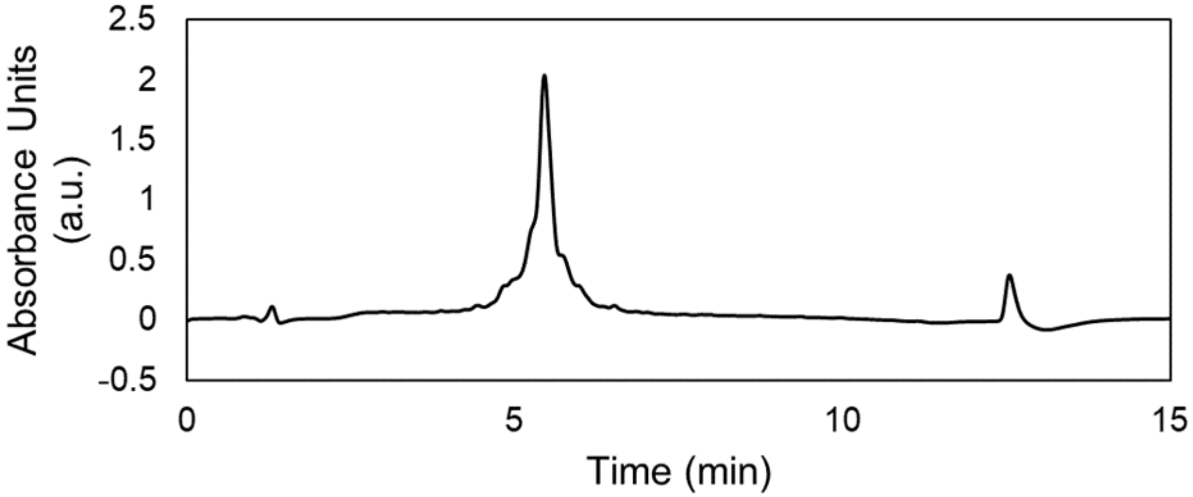

Figure S11. Analytical HPLC chromatogram of crude L-K_3^{11}. The peptide was eluted on a linear AB gradient from 5% to 95% B (v/v) over 9 minutes, where A is ultrapure water + 0.1% TFA and B is acetonitrile + 0.1% TFA.

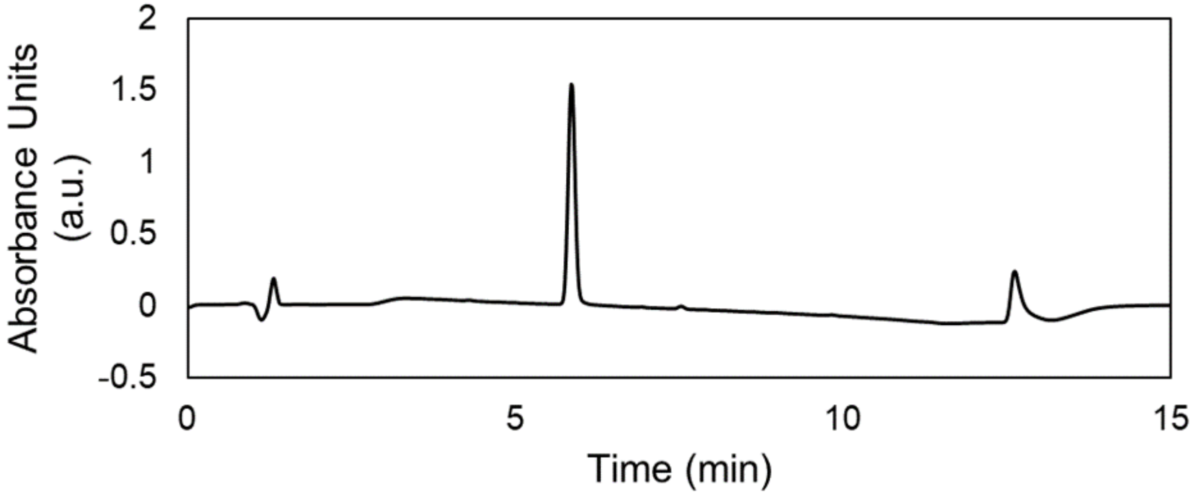

Figure S12. Analytical HPLC chromatogram of purified L-K_3^{11}. The primary peak corresponding to the purified peptide accounts for >99% of the total peak area. The peptide was eluted on a linear AB gradient from 5% to 95% B (v/v) over 9 minutes, where A is ultrapure water + 0.1% TFA and B is acetonitrile + 0.1% TFA.

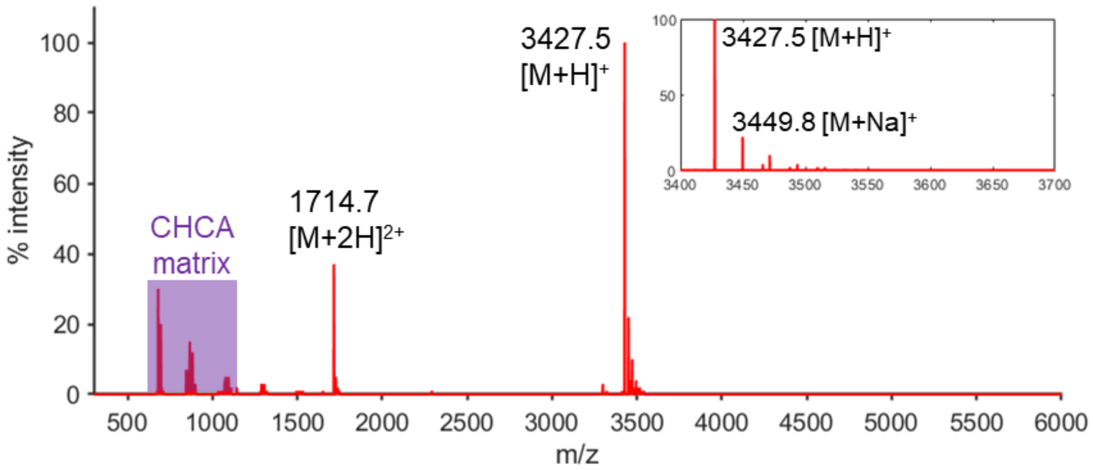

Figure S13. MALDI-TOF mass spectrum of purified L-K_3^{11}.

1.5 D-K_3^{11}

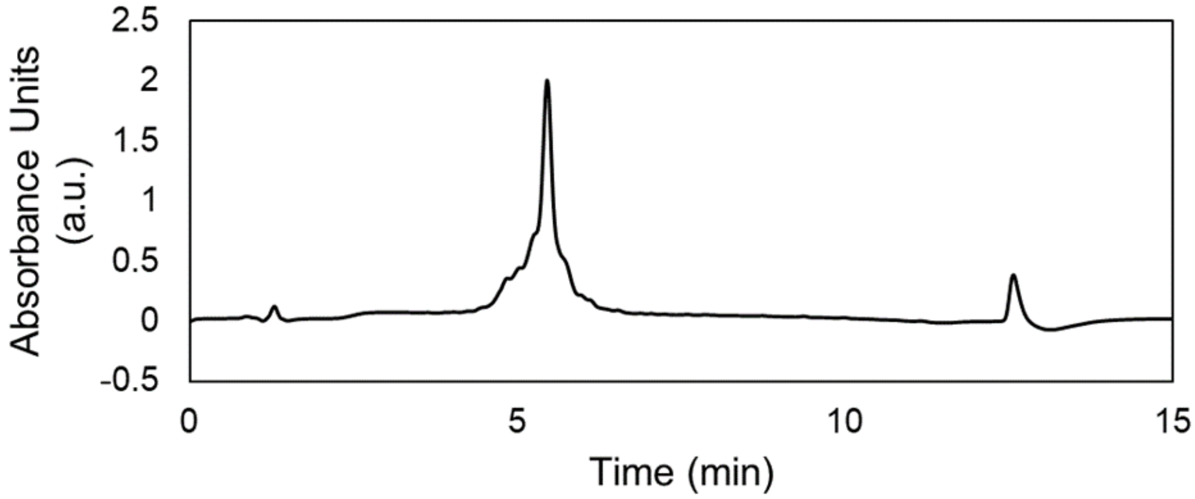

Figure S14. Analytical HPLC chromatogram of crude D-K_3^{11}. The peptide was eluted on a linear AB gradient from 5% to 95% B (v/v) over 9 minutes, where A is ultrapure water + 0.1% TFA and B is acetonitrile + 0.1% TFA.

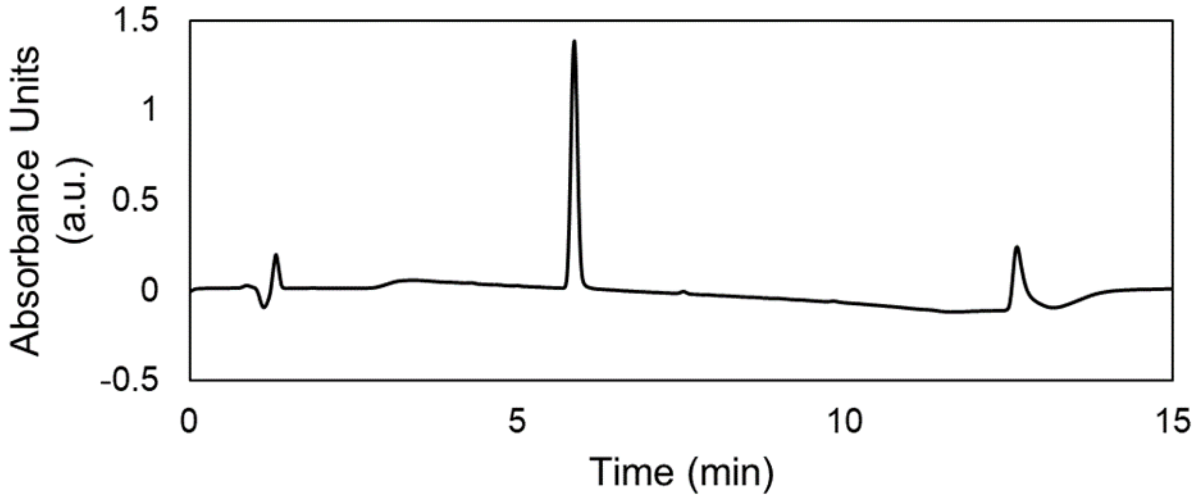

Figure S15. Analytical HPLC chromatogram of purified D-K_3^{11}. The primary peak corresponding to the purified peptide accounts for >99% of the total peak area. The peptide was eluted on a linear AB gradient from 5% to 95% B (v/v) over 9 minutes, where A is ultrapure water + 0.1% TFA and B is acetonitrile + 0.1% TFA.

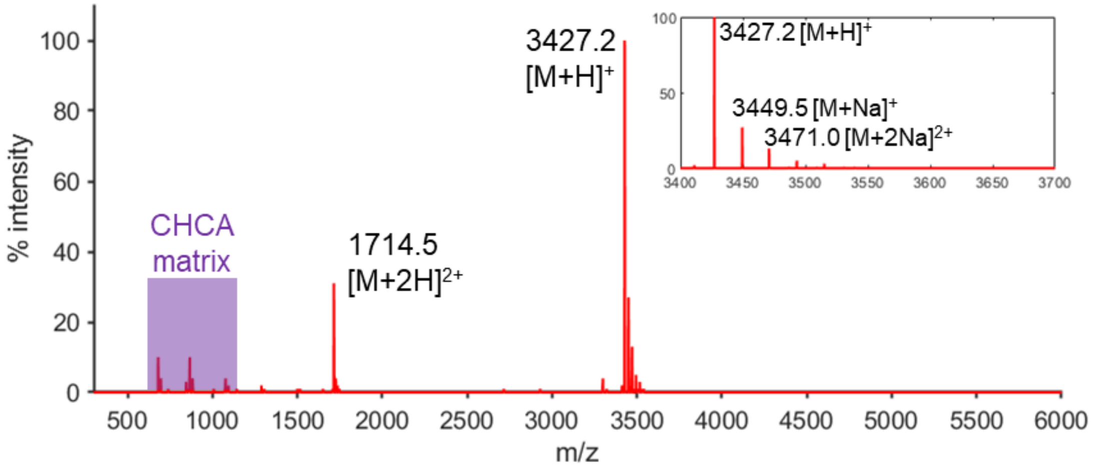

Figure S16. MALDI-TOF mass spectrum of purified D-K_3^{11}.

1.6 L-E_3^{11}

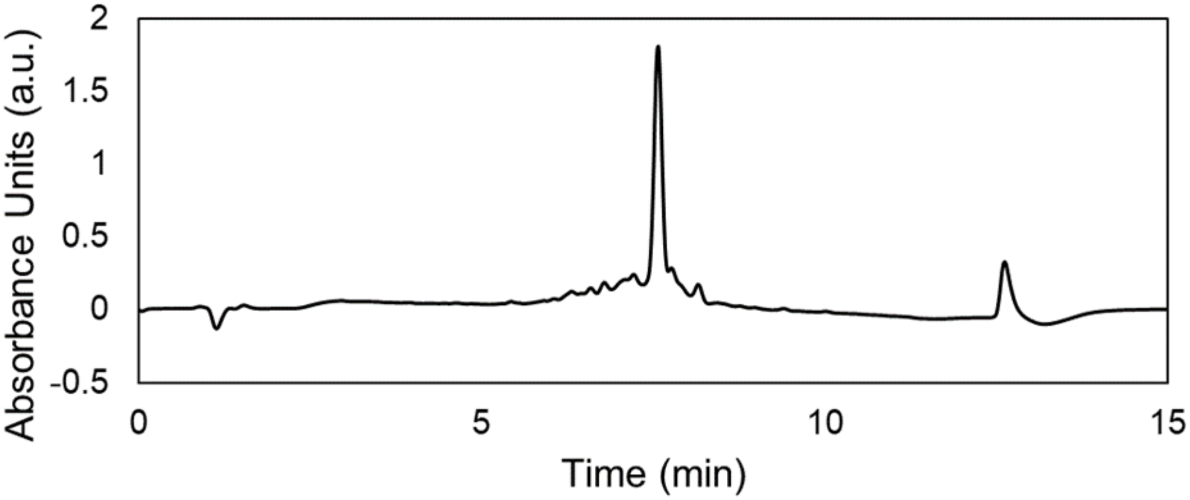

Figure S17. Analytical HPLC chromatogram of crude L-E_3^{11}. The peptide was eluted on a linear AB gradient from 5% to 95% B (v/v) over 9 minutes, where A is ultrapure water + 0.1% TFA and B is acetonitrile + 0.1% TFA.

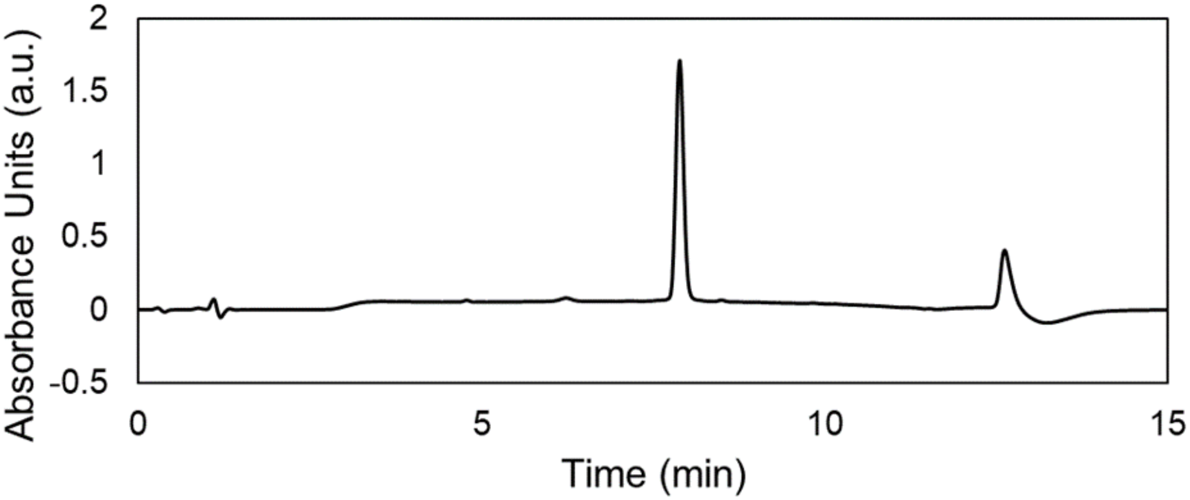

Figure S18. Analytical HPLC chromatogram of purified L-E_3^{11}. The primary peak corresponding to the purified peptide accounts for >97% of the total peak area. The peptide was eluted on a linear AB gradient from 5% to 95% B (v/v) over 9 minutes, where A is ultrapure water + 0.1% TFA and B is acetonitrile + 0.1% TFA.

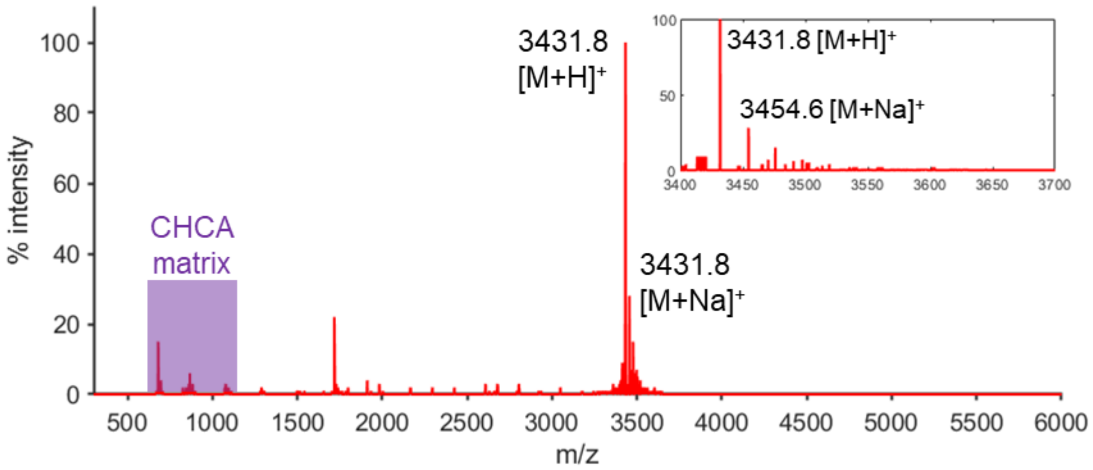

Figure S19. MALDI-TOF mass spectrum of purified L-E_3^{11}.

1.7 MALDI of CHCA matrix

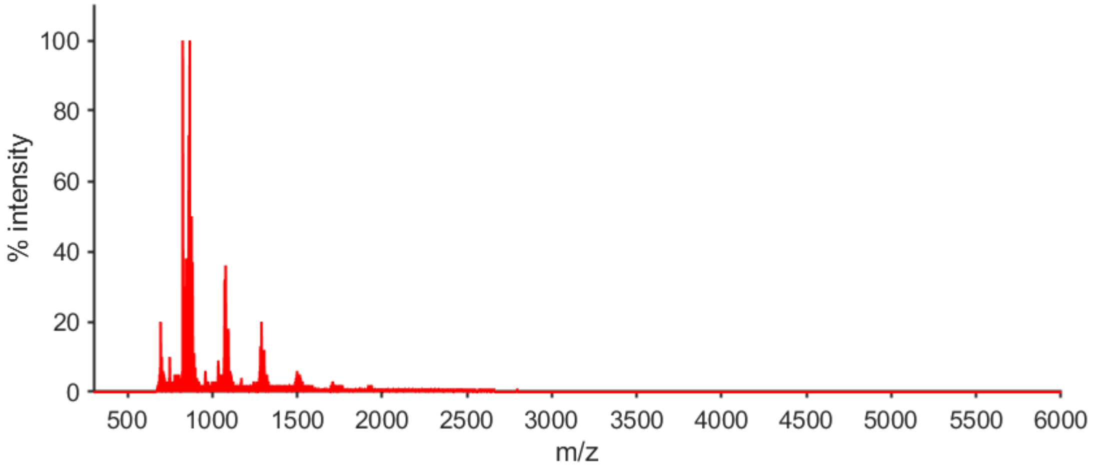

Figure S20. MALDI-TOF mass spectrum of CHCA matrix.

2. Additional ITC thermograms

2.1 Replicate of L-K_4^7 titrated into L-E_4^7

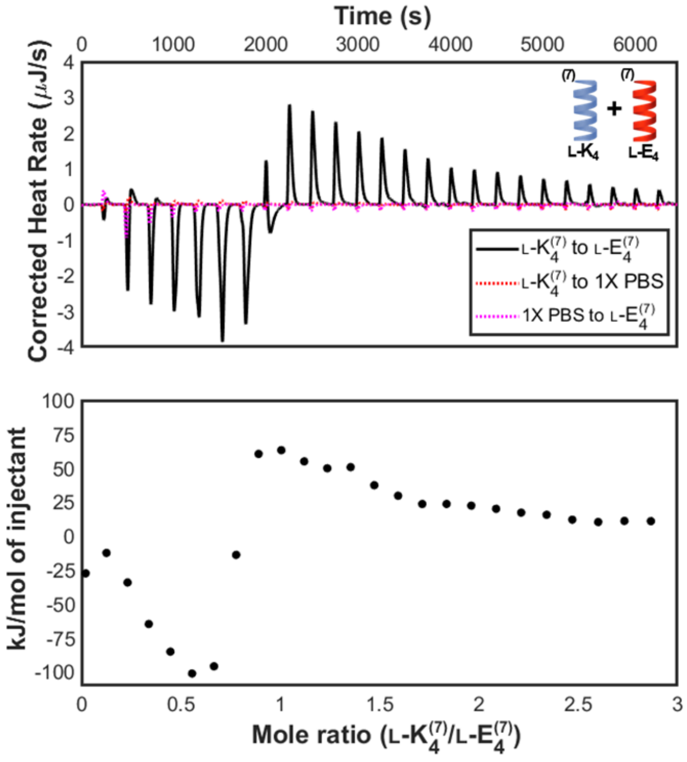

Figure S21. Thermogram and integrated binding heat from the titration of L-K_4^7 into L-E_4^7 at pH 7.4 in 1X PBS.

2.2 Replicate of D-K_4^7 titrated into L-E_4^7

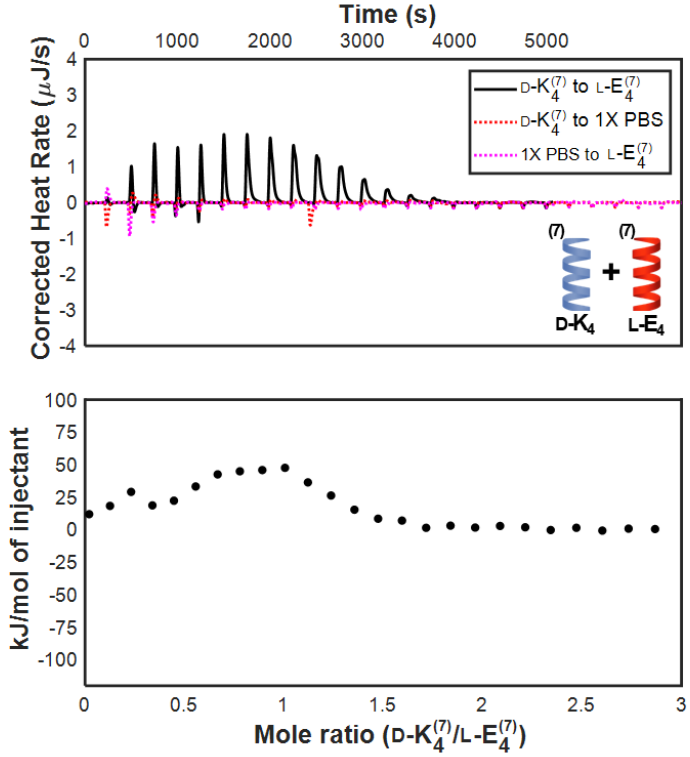

Figure S22. Thermograms and integrated binding heats from the titration of D-K_4^7 into L-E_4^7 at pH 7.4 in 1X PBS.

2.3 Replicate of L-K_3^{11} titrated into L-E_3^{11}

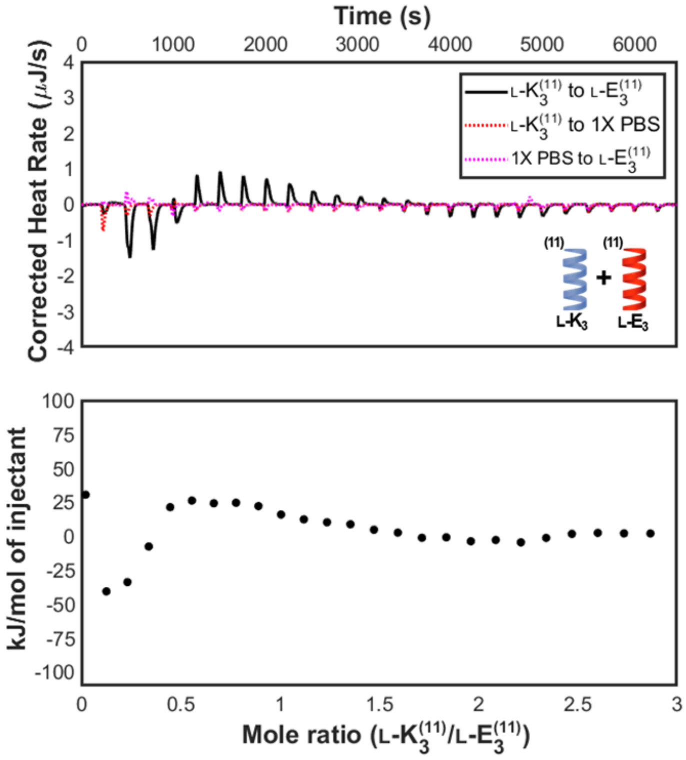

Figure S23. Thermograms and integrated binding heats from the titration of L-K_3^{11} into L-E_3^{11} at pH 7.4 in 1X PBS.

2.4 Replicate of D-K_3^{11} titrated into L-E_3^{11}

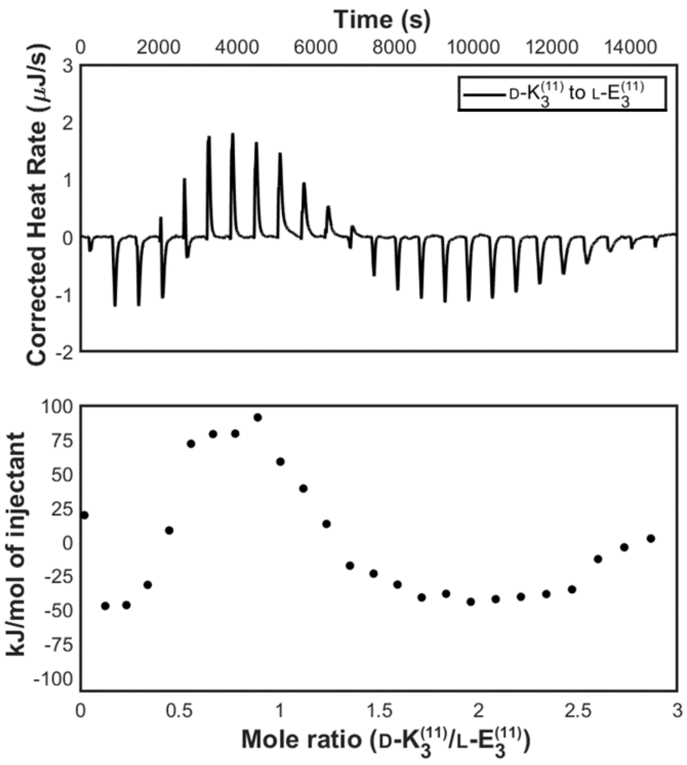

Figure S24. Thermogram and integrated binding heats from the titration of D-K_3^{11} into L-E_3^{11} at pH 7.4 in 1X PBS with 600 s spacing between injections.

3. CD spectra of coiled coils

3.1 Determination of CD spectroscopy conditions

The experimental parameters of cuvette path length, buffer concentration, and wavelength range were all varied to find the best experimental conditions for this system. There are three characteristic features of the CD spectrum of an α-helix, including (for an L-peptide), negative peaks at 222 nm and 208 nm as well as a positive peak at 193 nm. Therefore, we initially decided to collect data from 190 nm – 250 nm, choosing path length and buffer concentration based on obtaining quality data in this range. To this end, we observed the CD and high tension (HT) voltage signals for data collected in 1 mm and 0.1 mm cuvettes for 1X and 0.1X PBS. HT voltage is used to control the gain of the detector to obtain an ideal signal to noise ratio. When a sample absorbs too much light, the HT increases rapidly, and when HT is ≥ 700, the photons reaching the detector are not sufficient to provide reliable CD data. We found that, when using a path length of 1 mm and 1X PBS, the HT increased rapidly below 200 nm, leading to unreliable CD data, but for 0.1X PBS in a 1 mm cuvette or 1X PBS in a 0.1 mm cuvette, there were no problems with data reliability (**Figure S25**). Based on this data and in an attempt to keep the buffer concentration consistent with other experiments in this manuscript, we initially decided to use 1X PBS in a 0.1 mm cuvette

to take CD measurements. However, we were surprised to find that under these conditions for a 25 μM solution of L-K_4^7, the CD spectrum displayed none of the characteristic peaks for an α-helix (**Figure S26, red**). For comparison, we also obtained a CD spectrum of 25 μM L-K_4^7 in 1X PBS in a 1 mm cuvette and observed the characteristic peaks at 222 nm and 208 nm (**Figure S26, black**). We also obtained CD spectra of L-K_4^7 in 0.1X PBS in both 1 mm and 0.1 mm cuvettes (**Figure S26, green and blue**) and found that characteristic α-helical peaks were only observed in the 1 mm cuvette. These data indicate that CD signal depends on path length. For this reason, and to keep the buffer concentration consistent with other experiments, we decided to move forward using the 1 mm cuvette and 1X PBS for wavelengths from 200 nm – 250 nm, relying on the two characteristic α-helical peaks at 222 nm and 208 nm to demonstrate helicity despite not having reliable data for the characteristic peak at 193 nm.

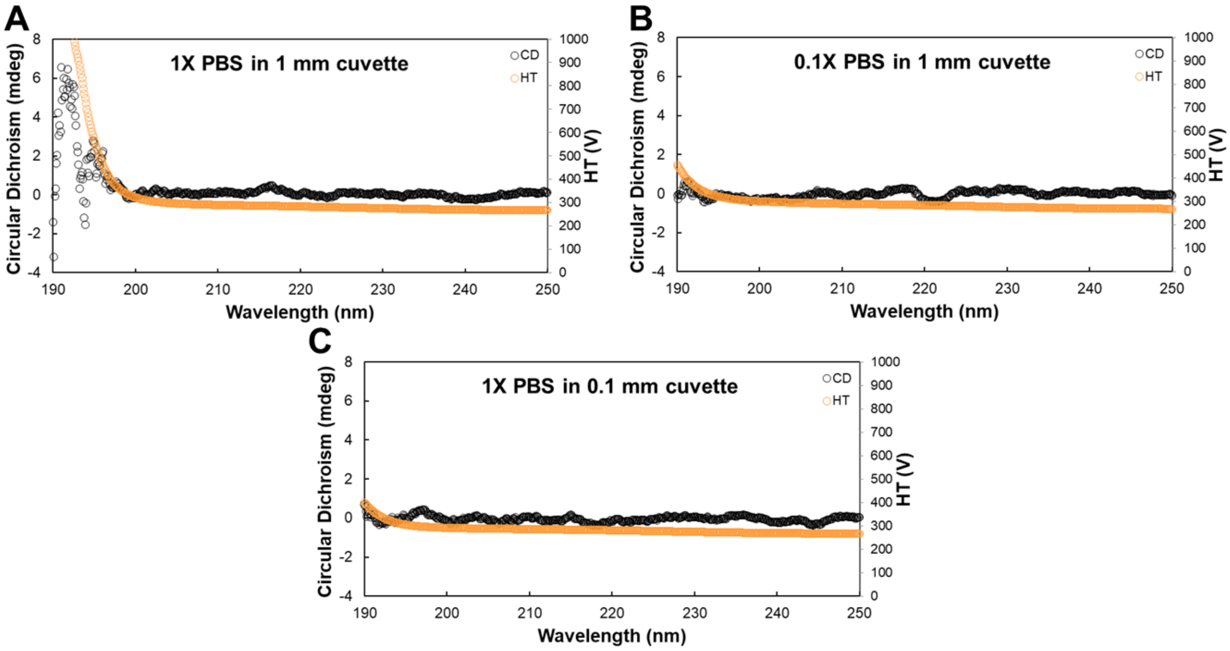

Figure S25. CD and HT voltage of A) 1X PBS in a 1 mm cuvette, B) 0.1X PBS in a 1 mm cuvette, and C) 1X PBS in a 0.1 mm cuvette. The HT voltage for the 1X PBS in a 1 mm cuvette increases rapidly between 200 nm – 190 nm, resulting in unreliable data. The HT voltage remains below 700 V for the other conditions, indicating that the data is reliable for the full range of 190 nm – 250 nm.

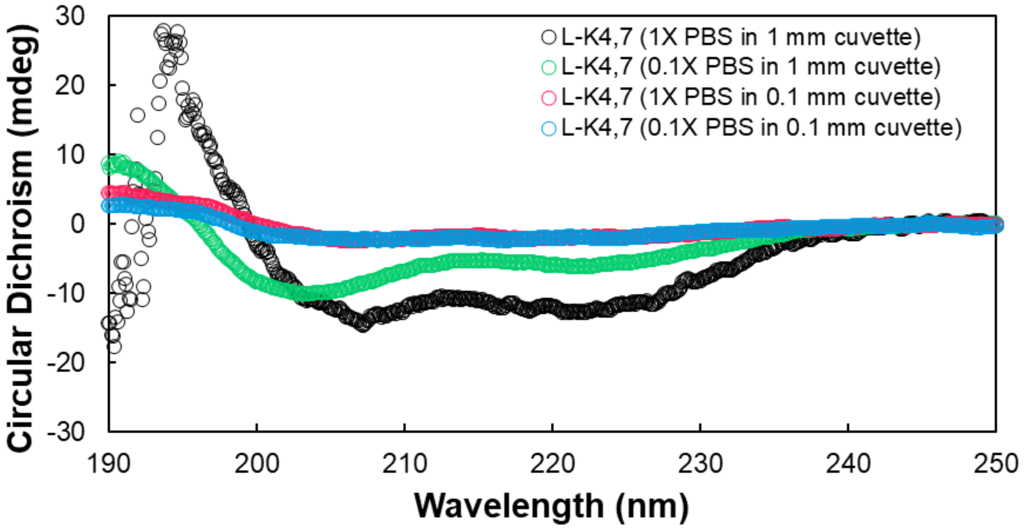

Figure S26. CD spectra of 25 µM L-K_4^7 in 1X PBS or 0.1X PBS measured in a 1 mm or 0.1 mm cuvette. Characteristic peaks for α-helices at 222 nm and 208 nm were only observed in samples measured in a 1 mm cuvette.

3.2 CD spectroscopy of L-K_4^7, L-E_4^7, D-K_4^7, L-K_3^{11}, L-E_3^{11}, and D-K_3^{11}

CD spectroscopy was used to confirm the secondary structure and stereochemistry of the coiled coils used in this study. All coiled coils were helical at 100 µM in 1X PBS (evidenced by peaks at 222 nm and 208 nm), while D-coils exhibit mean residue ellipticities > 0 and L-coils exhibit mean residues ellipticities < 0, consistent with the expected stereochemistry (**Figure S27**).

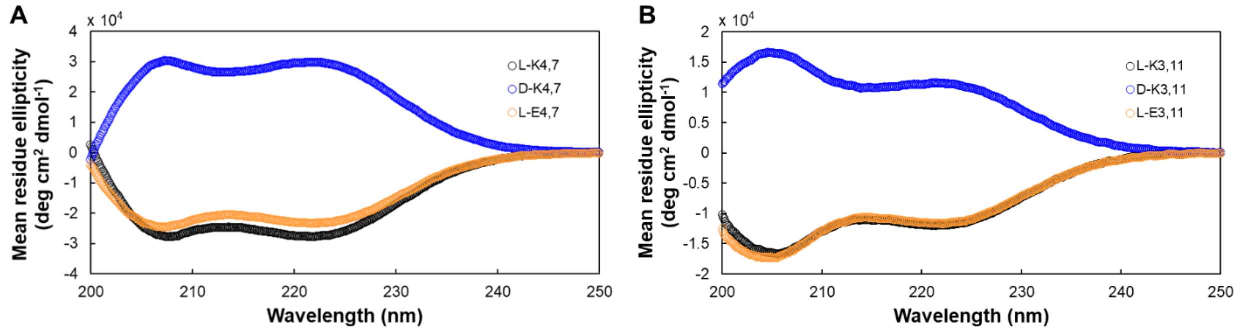

Figure S27. CD spectra of A) L-K_4^7, L-E_4^7, and D-K_4^7 and B) L-K_3^{11}, L-E_3^{11}, and D-K_3^{11}. All coils were helical and exhibited the expected stereochemistry.

3.3 CD spectroscopy of blended coiled coils

Upon mixing homochiral or heterochiral blends of heptads and hendecads, we used CD spectroscopy to compare the secondary structure of the blended coiled coils to the individual coiled coils (**Figure S28**). For homochiral blends, the coiled coils with a heptad repeating sequence were found to have a slightly greater mean residue ellipticity at 208 nm and 222 nm, the wavelengths most associated with α-helicity. The hendecad

coiled coils similarly exhibited greater mean residue ellipticities at 208 nm and 222 nm for homochiral blends. On the other hand, heterochiral blends of both heptad and hendecad coiled coils resulted in signals close to zero across the wavelength tested, due to the opposing stereochemistries of the blended peptides.

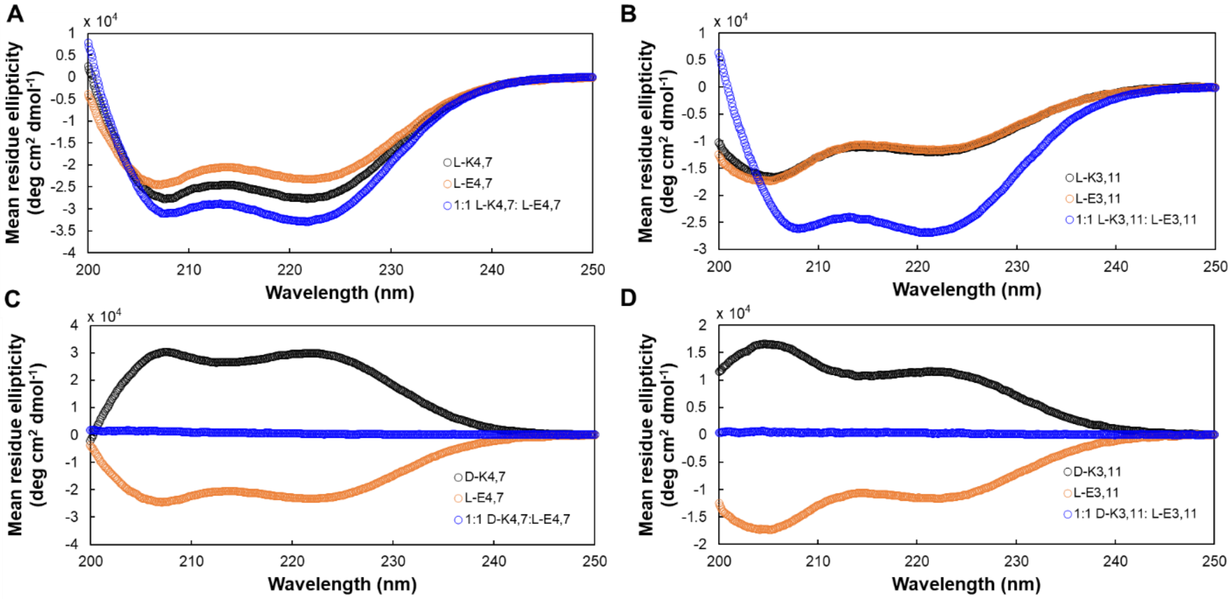

Figure S28. CD spectra of A) L-K_4^7, L-E_4^7, and 1:1 L-K_4^7: L-E_4^7, B) L-K_3^{11}, L-E_3^{11}, and 1:1 L-K_3^{11}: L-E_3^{11}, C) D-K_4^7, L-E_4^7, and 1:1 D-K_4^7: L-E_4^7, and D) D-K_3^{11}, L-E_3^{11}, and 1:1 D-K_3^{11}: L-E_3^{11}. All individual coils were helical as evidenced by peaks present at 208 nm and 222 nm. For the homochiral coiled coils, blending the heptad coiled coils (A) or hendecad coiled coils (B) resulted in a mixture with stronger helicity, as the mean residue ellipticity at these wavelengths was greater. Blending the heterochiral coiled coils (C and D) resulted in effectively no CD signal as the destructive interference of the equimolar peptides of opposing stereochemistry eliminates the signal.

3.4 Thermal stability of L-K_4^7: L-E_4^7, D-K_4^7: L-E_4^7, L-K_3^{11}: L-E_3^{11}, and D-K_3^{11}: L-E_3^{11} as measured by CD spectroscopy as a function of temperature

Temperature-dependent CD spectroscopy was used to assess the stability of homochiral and heterochiral heptad complexes. Solutions of 200 μM L-K_4^7, L-E_4^7, D-K_4^7, L-K_3^{11}, L-E_3^{11}, and D-K_3^{11} were prepared in 1X PBS, then equal volumes of L-K_4^7 and L-E_4^7, D-K_4^7 and L-E_4^7, L-K_3^{11} and L-E_3^{11}, or D-K_3^{11} and L-E_3^{11} were stirred together overnight to form an equimolar complex. CD spectra were taken of the complex solutions the next day from 5 °C to 90 °C in 5 °C increments. The homochiral heptads (**Figure S29 A**) and homochiral hendecads (**Figure S30 A**) maintained a helical conformation even at 90 °C, as evidenced by the retention of characteristic peaks at 222 nm and 208 nm, although the helical character of both complexes was reduced as measured by the increase in mean residue ellipticity at 222 nm (**Figure S29 A and Figure S30 A, bottom panel**). Unsurprisingly, the competing positive and negative CD signals of the D- and L-

hendecad coils led to a lack of signal for both heterochiral heptads and heterochiral hendecads (**Figure S29 B and Figure S30 B**).

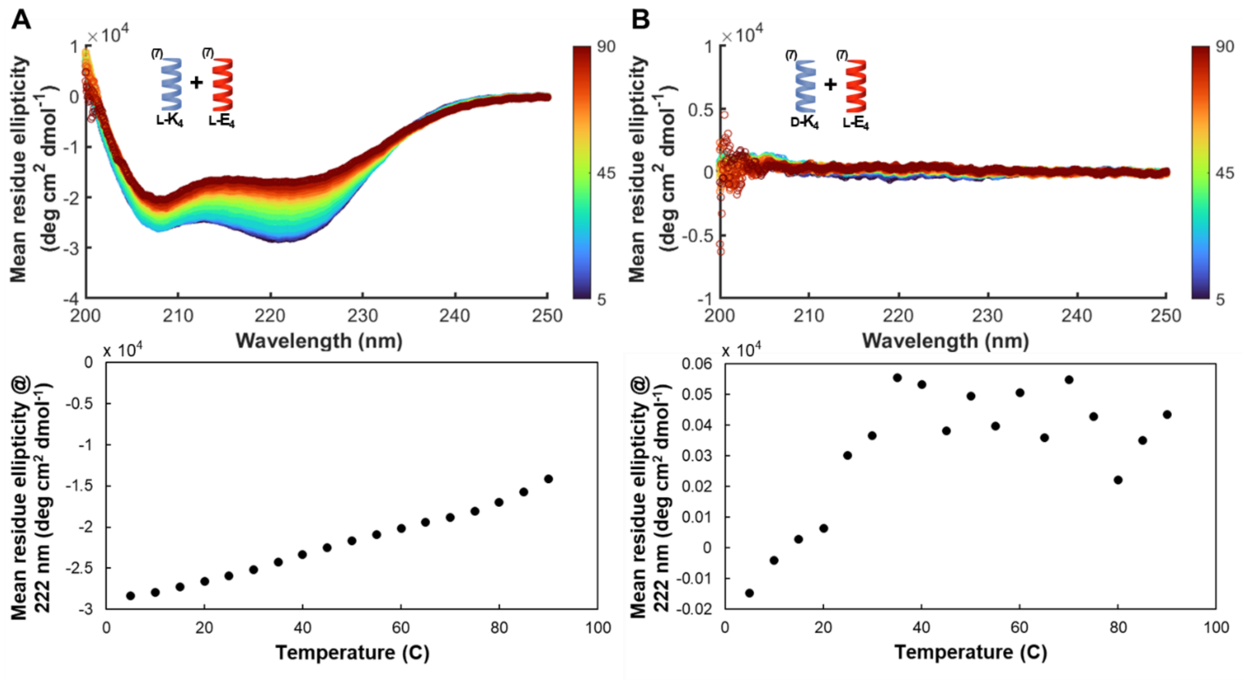

Figure S29. CD spectra as a function of temperature for A) L-K_4^7: L-E_4^7 and B) D-K_4^7: L-E_4^7. The top panel shows all CD spectra from 5 °C to 90 °C in intervals of 5 °C and the bottom panel shows just the mean residue ellipticity of the complex at 222 nm as a measure of helicity.

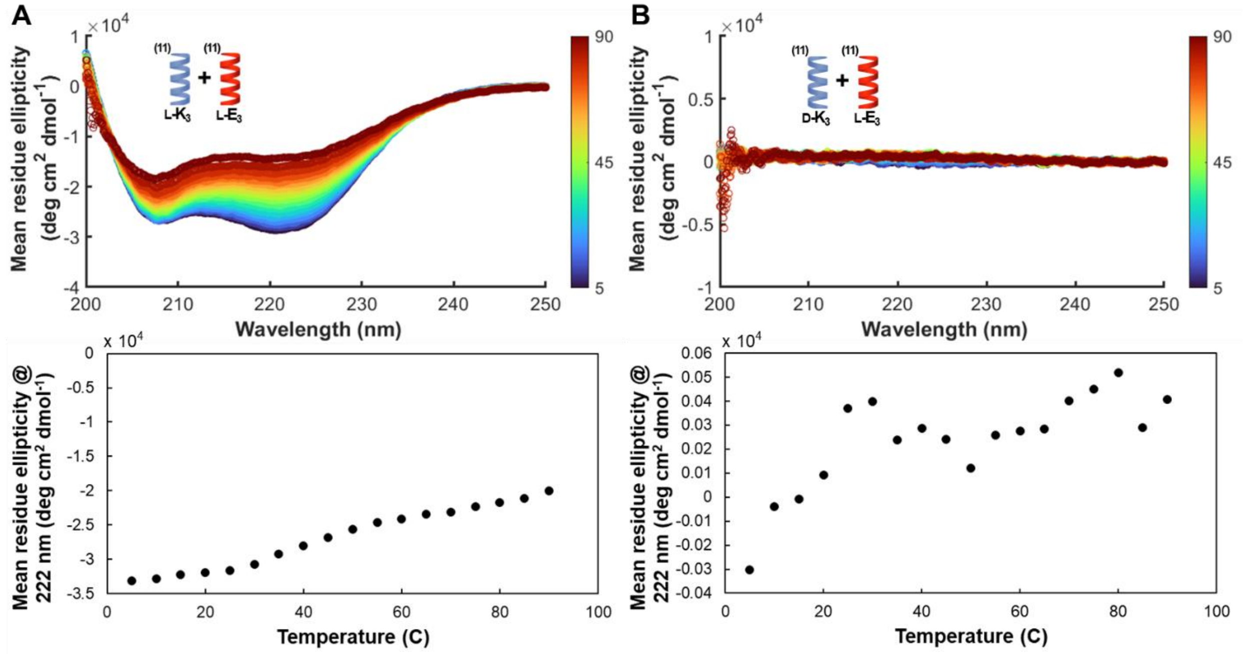

Figure S30. CD spectra of A) L-K_3^{11}: L-E_3^{11} and B) D-K_3^{11}: L-E_3^{11}. The top panel shows all CD spectra from 5 °C to 90 °C in intervals of 5 °C and the bottom panel shows just the mean residue ellipticity of the complex at 222 nm as a measure of helicity.

4. Additional degradation data

4.1 Degradation of coiled coils in the absence of Proteinase K

As a control experiment, coiled coil complexes were incubated in 1X PBS following the same procedure as the degradation experiment without adding Proteinase K to test their stability in buffer. For heptad complexes (L-K_4^7: L-E_4^7 and D-K_4^7: L-E_4^7) and hendecad complexes (L-K_3^{11}: L-E_3^{11} and D-K_3^{11}: L-E_3^{11}), the complexes remained stable out to 24 or 30 h (**Figure S31 and Figure S32**).

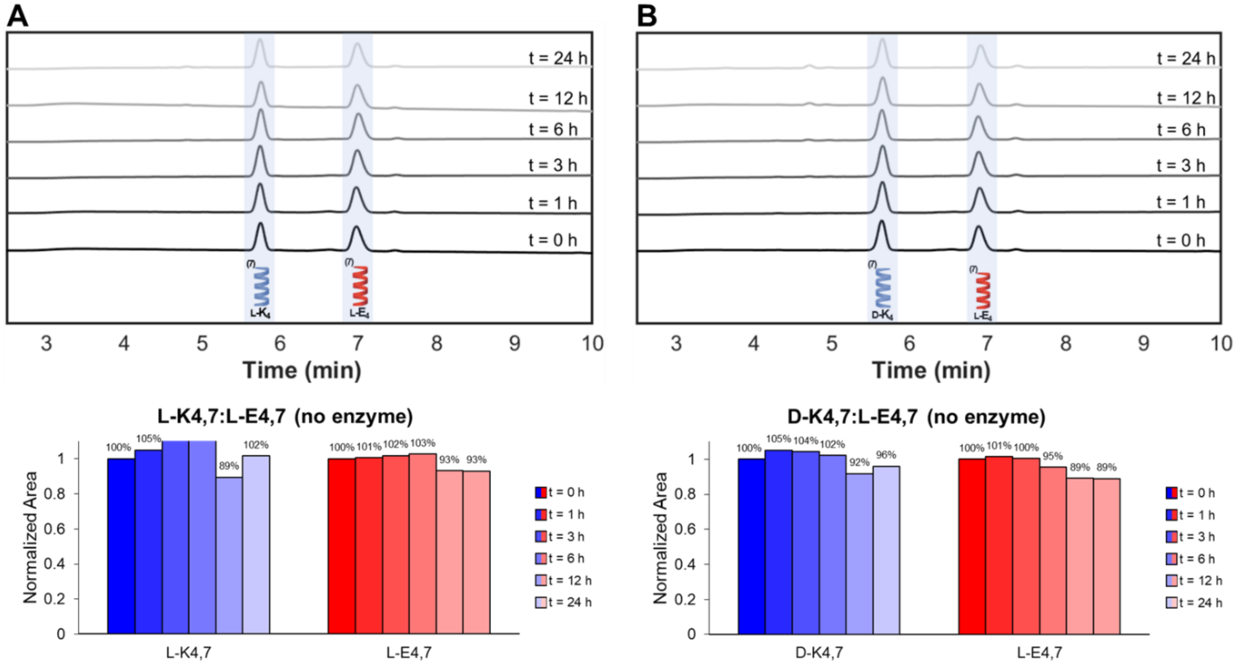

Figure S31. Stability of A) L-K_4^7: L-E_4^7 and B) D-K_4^7: L-E_4^7 upon incubation in 1X PBS.

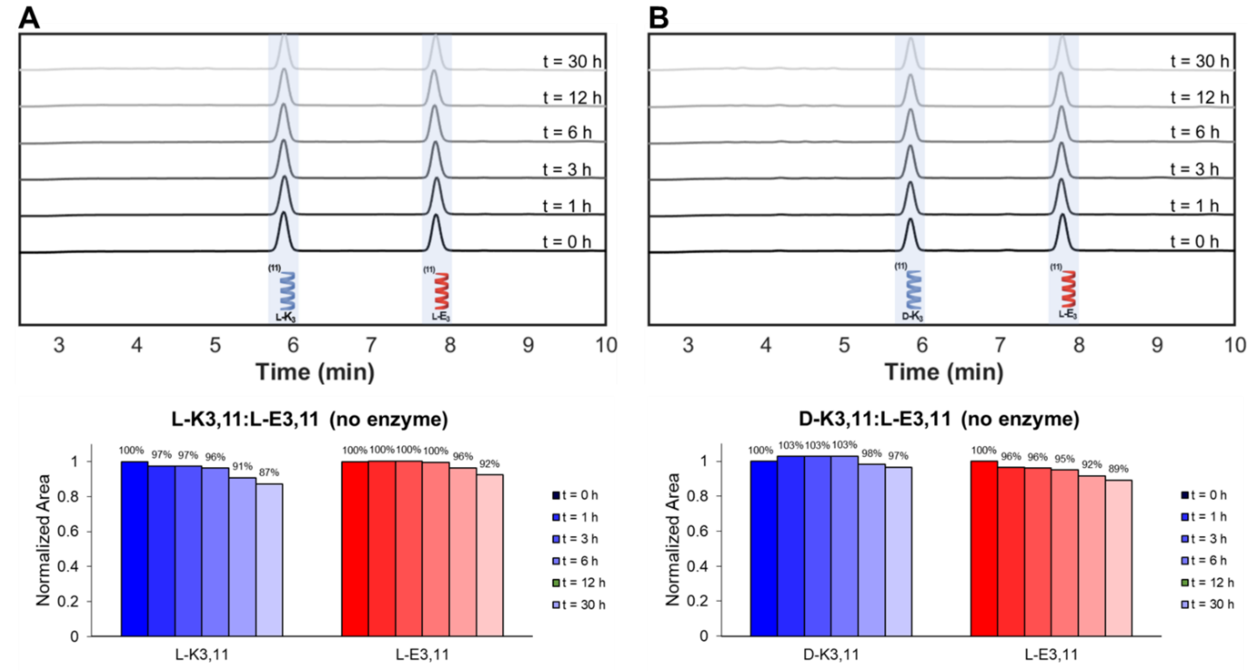

Figure S32. Stability of A) L-K_3^{11} : L-E_3^{11} and B) D-K_3^{11} : L-E_3^{11} upon incubation in 1X PBS.

4.2 Extended degradation of D-K_3^{11}: L-E_3^{11} upon incubation with Proteinase K

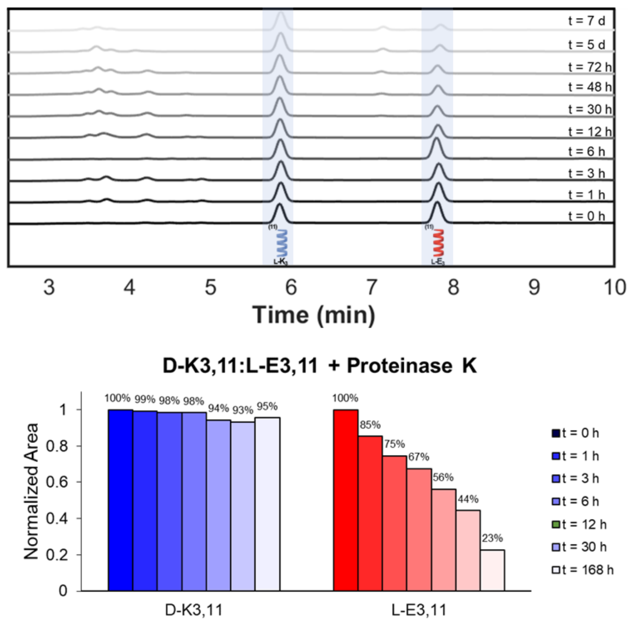

Figure S33. Proteolytic stability of D-K_3^{11}: L-E_3^{11} in the presence of 5 µg/mL Proteinase K. HPLC chromatograms and percent intact K_3^{11} and E_3^{11} by peak area immediately after addition of and upon incubation for 1, 3, 6, 12, 30, 48, 72, 120, and 168 h with Proteinase K.

5. MATLAB script to normalize elution time for degradation data

```
clear all
clc

% This code is intended to "normalize" HPLC data by lining up a peak in the plot at
the same x value for the entire set of data.  For example, I am
% creating this because I have degradation data, but the peaks shift slightly in
elution time for each time point run.  I want to align all of
% them so that the peak associated with one peptide always elutes at the same time.

% We start by reading in the data from a text file.
T = readtable('hplcdata.txt');
x(1,:) = transpose(table2array(T(1:height(T), 1)));
y(1,:) = transpose(table2array(T(1:height(T), 2)));
for i = 1:width(T)-1
    x(i,:) = x(1,:);
end
for j = 2:width(T)
    y(j-1,:) = transpose(table2array(T(1:height(T), j)));
end

% earlytime and latetime set the bounds for where the code will search for a peak in
the data to align.  For example, I have a peak that elutes at
% ~5.8 min in every run, so I have the code search for the max value between the
times of 5.5 min and 6.5 min.
earlytime = 5.5;
latetime = 6.5;

% Here, we crop the datasets to work with only the data between earlytime and
latetime.  However, often the xdata (Time) from HPLC does not include
% whole numbers, so I search for any value that is without 0.001 of earlytime and
latetime in the xdata and crop the datasets to that.
croppedx = x(1,find(x(1,:)>earlytime-0.001 &
x(1,:)<earlytime+0.001):find(x(1,:)>latetime-0.001 & x(1,:)<latetime+0.001));
for k = 1:width(T)-1
    croppedy(k,:) = y(k,find(x(1,:)>earlytime-0.001 &
x(1,:)<earlytime+0.001):find(x(1,:)>latetime-0.001 & x(1,:)<latetime+0.001));
end

% This code only cares about the index, or position, of the max value and doesn't
need the actual value, so we use the ~ operator to discard the
% actual value, and I0-I# to store the index.  For cases where a dataset doesn't have
a peak in the region of interest (bewteen earlytime and
% latetime), we just set the index to I(1) (giving it the same index as the first
dataset, meaning we won't shift the data). This is because we don't
% want to shift the data based on random noise in the baseline.  The noise value can
be set based on your data.
noise = 0.09;
for l = 1:width(T)-1
    if max(croppedy(l,:)) > noise
        [~,I(l)] = max(croppedy(l,:));
    else
        I(l) = I(1);
    end
```

```
end

% This is the code block that actually shifts the data.  We know the index of the
peak in the first dataset that we want to align the others to
% (I0), so we take that x value and subtract the x value of the peak in the next
dataset.  If the peaks elute at the same time, this value is 0.  If
% they elute at slightly different times, the value of (croppedx(I0)-croppedx(I#))
will be the value in x that the dataset needs
% to move.  We change the values of the x data for each dataset individually.
for m = 2:width(T)-1
    x(m,:) = x(m,:) + (croppedx(I(1))-croppedx(I(m)));
end

% To stack the data vertically, I am simply adding value to each subsequent dataset.
y0 is plotted as is, y1 is plotted 1.5 y units above that, and
% so on.
for n = 2:height(y)
    y(n,:) = y(n,:) + 1.5*(n-1);
end

% Plot the data!
cmap = gray(height(y)+1);
for t = 1:height(y)
    plot(x(t,:),y(t,:), 'LineWidth', 1.75); axis([2.5 10 -3 9])
    hold on
end
% Modify the plot to make it look nicer
ax = gca;
ax.TickLength = [0,0];
ax.LineWidth = 2;
ax.FontName = 'Arial';
ax.FontSize = 16;
yticks([]);
xlabel('\bf Time (min)', 'Fontsize', 18);
w = 8;
h = 4;
set(gcf,'units','inches','position',[4,4,w,h]);
%legend('t = 0 h', 't = 1 h', 't = 3 h', 't = 6 h', 't = 12 h', 't = 30 h', 't = 48
h', 't = 72 h', 't = 120 h', 't = 168 h', 't = 240 h', 'Location', 'North',
'Orientation','horizontal', 'FontSize', 11);
%legend boxoff
colororder(cmap);
```
